# A population pharmacokinetic model library for polymyxin B: clinical implementation of model-informed precision dosing

**DOI:** 10.3389/fphar.2026.1900585

**Published:** 2026-08-25

**Authors:** Shimin Wang, Xiaoyan Cui, Lili Li, Lijie Kang, Guangjie Hou

**Affiliations:** 1 Department of Pharmacy, Fuyang Normal University Second Affiliated Hospital, Fuyang, China; 2 Science and Education Department, Fuyang Normal University Second Affiliated Hospital, Fuyang, China

**Keywords:** model library, model-informed precision dosing, pharmacometrics, polymyxin B, population pharmacokinetic modeling

## Abstract

**Introduction:**

Polymyxin B remains a key last-resort therapy for infections caused by multidrug-resistant Gram-negative bacteria. Nevertheless, its substantial pharmacokinetic (PK) variability and narrow therapeutic window pose significant challenges to dose individualization. Despite the availability of multiple published population pharmacokinetic (popPK) models, a comprehensive repository to enable model-informed precision dosing has not yet been established. Accordingly, this study aimed to develop a comprehensive library of polymyxin B popPK models to facilitate individualized treatment in clinical practice.

**Methods:**

A systematic review was performed to locate published population pharmacokinetic models of polymyxin B. Three bibliographic databases, namely, PubMed, Web of Science, and Embase, were searched through November 2025 without a lower date limit. For each eligible study, information was collected on study design, administered doses, blood-sampling schemes, structural model characteristics, parameter values, and covariate effects. Articles published in languages other than English, duplicate records, and studies based on nonparametric modeling approaches were excluded. The model database was constructed in R with the mrgsolve package, and an interactive Shiny platform was created to support exposure simulation and individualized model-informed dose prediction.

**Results:**

Twenty-two studies were incorporated into the polymyxin B model repository. The study populations included patients receiving extracorporeal membrane oxygenation (two studies), patients undergoing continuous renal replacement therapy or related renal replacement modalities (four studies), and patients with severe burns, liver dysfunction, lung or renal transplantation, obesity, renal impairment, or advanced age, as well as healthy volunteers. The repository also incorporated subpopulations with clinically relevant covariates known to influence PK, such as renal function and obesity. Most polymyxin B popPK models were described by a two-compartment model with linear elimination. In 10 of the 22 studies, renal function metrics were found to significantly impact distribution or elimination PK parameters. Internal validation was reported for all included models, whereas only two studies evaluated model performance using external datasets. Exposure simulations showed marked differences across models, indicating that fixed standard dosing regimens may have limited ability to consistently achieve target therapeutic concentrations.

**Conclusion:**

By harmonizing published population pharmacokinetic models in a single framework, this repository enables systematic exploration of model-based predictions across clinically relevant populations and may facilitate individualized polymyxin B therapy. However, given the complex clinical scenarios in critically ill patients, the heterogeneity of their physiological and pathological states, and the substantial interindividual PK variability of polymyxin B, further investigations are warranted to establish a more robust evidence base for precision dosing.

## 1 Introduction

As multidrug-resistant Gram-negative pathogens, including *Pseudomonas aeruginosa*, *Acinetobacter* baumannii, and *Klebsiella pneumoniae*, become increasingly prevalent, the clinical utility of conventional first-line antibiotics for severe infections has been progressively undermined by escalating antimicrobial resistance. Polymyxins have re-emerged as an important therapeutic option for multidrug-resistant Gram-negative infections, not only because of their unique mechanism of action targeting lipopolysaccharide, but also because they often retain antibacterial activity against pathogens resistant to most other available agents. In particular, polymyxin B (PMB) has regained a central role as a last-line therapy for these life-threatening infections (Jean et al., 2022; Li et al., 2024; Peng et al., 2025). Although PMB has substantial clinical value against multidrug-resistant (MDR) pathogens, its clinical use remains constrained by considerable toxicity, particularly nephrotoxicity and neurotoxicity, as well as a narrow therapeutic window (Nang et al., 2021; Peng et al., 2025). The inherent toxicity of PMB, together with its pronounced pharmacokinetic (PK) variability, especially in critically ill patients, poses major challenges for individualized therapy. Although guideline-recommended dosing is based on body weight, fixed-dose regimens remain common in real-world practice because weight-based dosing is not always easily implemented. Moreover, the clinically relevant covariates responsible for PK variability have not been fully established and remain controversial across studies. As a result, conventional empirical dosing may not reliably achieve target exposure, with underexposure potentially leading to therapeutic failure and overexposure increasing the risk of toxicity (Tam et al., 2020; Zamri et al., 2025).

For systemic therapy, PMB is administered primarily by intravenous infusion. Following a 0.75 mg/kg intravenous infusion of PMB in healthy volunteers, the peak plasma concentration was 4.66 ± 0.46 mg/L, the area under the concentration-time curve from 0 to 24 h (AUC_0-24_) was 21.8 ± 1.83 mg h/L, and the elimination half-life was 5.44 ± 0.74 h (Liu et al., 2021). The major active components of PMB are polymyxin B1 (PMB1), polymyxin B2 (PMB2), polymyxin B3 (PMB3), and isoleucine-polymyxin B1 (Ile-PMB1), with no appreciable differences in pharmacokinetic characteristics or antibacterial activity among these components. In animal models, the distribution of PMB in muscle, heart, and liver was similar to that in blood. The kidney showed the highest tissue-to-serum concentration ratio (approximately 7 at 3 h and 18 at 6 h), followed by the lung (approximately 3 at 3 h and 2 at 6 h) (Manchandani et al., 2016). However, PMB exposure in the epithelial lining fluid was low (He et al., 2013).

International consensus guidelines recommend an AUC_ss,24h_ target of approximately 50–100 mg h/L for PMB, equivalent to a steady-state plasma concentration (Css) of 2–4 mg/L (Tsuji et al., 2019). Exposure below this range may compromise therapeutic efficacy and favor resistance development, whereas excessive exposure increases the likelihood of toxicity. In routine clinical practice, PMB dosing is commonly guided by actual or lean body weight; however, fixed-dose regimens are still used in some settings and may contribute to pharmacokinetic variability. Such variability, together with uncertainty in exposure and concerns about toxicity, may discourage the use of PMB in clinical practice, potentially delaying appropriate treatment and causing some patients to miss the optimal therapeutic window.

The 2024 Infectious Diseases Society of America (IDSA) guidance on the treatment of antimicrobial-resistant Gram-negative infections emphasizes the need to optimize dosing regimens according to pharmacokinetic/pharmacodynamic (PK/PD) principles (Tamma et al., 2024). Against the backdrop of the escalating burden of carbapenem-resistant organisms (CRO), clinical application of PK/PD principles provides a practical approach to maximize the therapeutic benefit of PMB (Liu S. et al., 2023).

Population pharmacokinetics (popPK) was developed to characterize pharmacokinetic variability attributable to intrinsic factors, such as physiological, pathological, and genetic characteristics, and extrinsic factors, such as concomitant therapies and clinical interventions. By quantifying the influence of these factors on drug exposure, popPK supports dose optimization, improves the likelihood of PK target attainment, and thereby enables individualized therapy. By integrating popPK models with pharmacodynamic targets and applying Bayesian estimation, dosing regimens can be optimized to enable individualized therapy (Oda et al., 2023). Over the past two decades, particularly in recent years, numerous popPK studies of PMB have been reported. These studies have generated a large number of models in a variety of patient populations, such as critically ill patients, patients with severe infections, obese patients, and patients with impaired renal function. Most models, particularly those based on rich sampling data, were characterized by a two-compartment structure. Many have undergone comprehensive validation, and some have been evaluated together with limited sampling strategies, showing encouraging utility for clinical implementation. The contribution of renal function to PMB disposition remains debated; however, several popPK analyses have identified renal markers as potentially relevant predictors of exposure. At the same time, dose individualization according to body weight has become more widely adopted in clinical practice.

Earlier reviews of PMB population pharmacokinetic evidence may not have captured all relevant models because of restricted search scopes and publication periods. In addition, few studies have systematically compared exposure predictions generated by different published models. To fill these gaps, the present study collected and evaluated available PMB popPK models, summarized covariates related to variability in drug disposition, and compared model-derived pharmacokinetic profiles under standardized simulation conditions. We also constructed an integrated PMB popPK model library to facilitate subsequent simulation, validation, and clinical application. In parallel, a Shiny-based application was created to support model-based exposure prediction and individualized dosing exploration. The application enables clinicians and pharmacists with limited programming expertise to modify patient-specific covariate profiles and dosing strategies, estimate the expected exposure of a given patient under alternative dosing regimens, compare model-predicted exposure across clinically relevant scenarios, and identify the most appropriate models for decision-making. This framework is designed to improve the accuracy and facilitate the clinical implementation of model-informed precision dosing (MIPD).

## 2 Methods

### 2.1 Search strategy

PubMed, Web of Science, and Embase were searched for population pharmacokinetic studies of PMB from database inception to November 2025. The search combined “polymyxin B” with terms related to population pharmacokinetics, including “population pharmacokinetics,” “pharmacokinetic model,” “population PK,” “popPK,” “PPK,” “nonlinear mixed-effects model,” and “NONMEM.” The search strategy and reporting followed the Preferred Reporting Items for Systematic Reviews and Meta-Analyses (PRISMA) guideline. Two authors independently and sequentially screened the records. Disagreements were resolved by adjudication from a third senior investigator.

Studies were included if they met the following criteria: (1) the study population comprised humans; (2) a population pharmacokinetic analysis was performed; (3) the model was a parametric nonlinear mixed-effects model; and (4) the article was published in English.

Studies were excluded if they met any of the following criteria: (1) *in vitro* or animal studies; (2) methodological studies, reviews, case reports, or conference abstracts/proceedings; (3) models developed using nonparametric approaches; or (4) insufficient reporting of the popPK and/or PD parameters required for model reconstruction.

### 2.2 Data collection and coding

For each eligible publication, two reviewers independently recorded predefined variables using a standardized extraction form. The extracted items comprised three domains: first, cohort-level information, including geographic region, age, body weight, sex distribution, and relevant laboratory measurements; second, study and treatment details, such as dosing strategy, sample size, analytical assay, and lower limit of quantification; and third, population pharmacokinetic model information, including structural equations, parameter estimates, model specification, evaluated covariates, covariate selection procedures, between subject variability, between-occasion variability, and unexplained residual error.

### 2.3 Literature quality assessment

Following quality assessment approaches used in previous systematic reviews of population pharmacokinetic studies (Liu et al., 2023b; Ju et al., 2024; Ju et al., 2025), we evaluated the methodological and reporting quality of the included studies using a checklist comprising five domains and 32 items (Supplementary Table S2). The checklist focused on core information needed to describe and interpret clinical pharmacokinetic analyses. Each item was scored as 1 when fully reported, 0.5 when partially reported, and 0 when absent. For each popPK study, an overall compliance percentage was then derived according to the formula below:

$$Compliance=\left(\frac{\text{sum}\;\text{of}\;\text{items}\;\text{reported}}{\text{sum}\;\text{of}\;\text{all}\;\text{items}}\right)\times 100\%$$

### 2.4 Comparison of covariate effects across studies

We summarized covariates associated with clearance (CL) and volume of distribution (Vd) across the retrieved population pharmacokinetic models. Because clearance is the primary determinant of maintenance dosing and systemic exposure, subsequent comparisons focused on covariate effects on PMB CL. To allow comparison among studies, continuous covariates were mapped onto a unified scale, whereas categorical covariates were represented by indicator values, with 0 denoting absence and 1 denoting presence of the factor.

For each study, the reported covariate range was used to derive the lower and upper predicted CL values. The median covariate value was aligned with the model-specific reference CL and served as the anchor for estimating the magnitude of covariate influence. Effects were then quantified as relative changes in CL and expressed as percentages of the corresponding study-specific CL span. These effects were summarized graphically with forest plots to enable cross-study comparison. The magnitude of covariate influence was derived using the equation below:

$$\text{Covariate effect}=\left(\frac{\text{The}\;\text{minimum}\;\text{CL}\;\text{or}\;\text{the}\;\text{maximum}\;\text{CL}}{\text{Reference}\;\text{CL}}\right)\times 100\%$$

### 2.5 Monte Carlo simulation

Monte Carlo simulation was used to examine model behavior and generate simulated PMB concentration profiles together with visual predictive distributions (VPDs). Published models were assumed to adequately describe the underlying data. Accordingly, the simulated predictive distributions of PMB concentrations were taken to reflect the original observations and their key features. Ordinary differential equation-based models were solved and simulated using the mrgsolve package (v1.1.1). For each included study, 1,000 virtual patients were simulated, and concentration-time curves were generated in R (v4.5.2) based on the corresponding population pharmacokinetic model.

### 2.6 Model simulation software

The Shiny package (v1.13.0) was combined with mrgsolve to build a PMB model knowledge base and embed it within an interactive simulation platform. Users can input demographic and clinical characteristics, including age, sex, and body weight or lean body weight, choose a candidate model, and define alternative dosing schedules. Exposure predictions were generated through Bayesian simulation around typical population parameter values, and uncertainty was summarized using prediction intervals incorporating the interindividual variability reported for each source model.

## 3 Results

### 3.1 Study selection

The literature search identified 1,169 records, of which 490 were removed as duplicates. After title and abstract screening based on predefined eligibility criteria, 46 articles were selected for full-text review. Ultimately, 22 studies fulfilled all inclusion criteria and were retained for the final analysis. The screening process is illustrated in Supplementary Figure S1, and the database-specific search queries are summarized in Supplementary Table S1.

**FIGURE 1:**
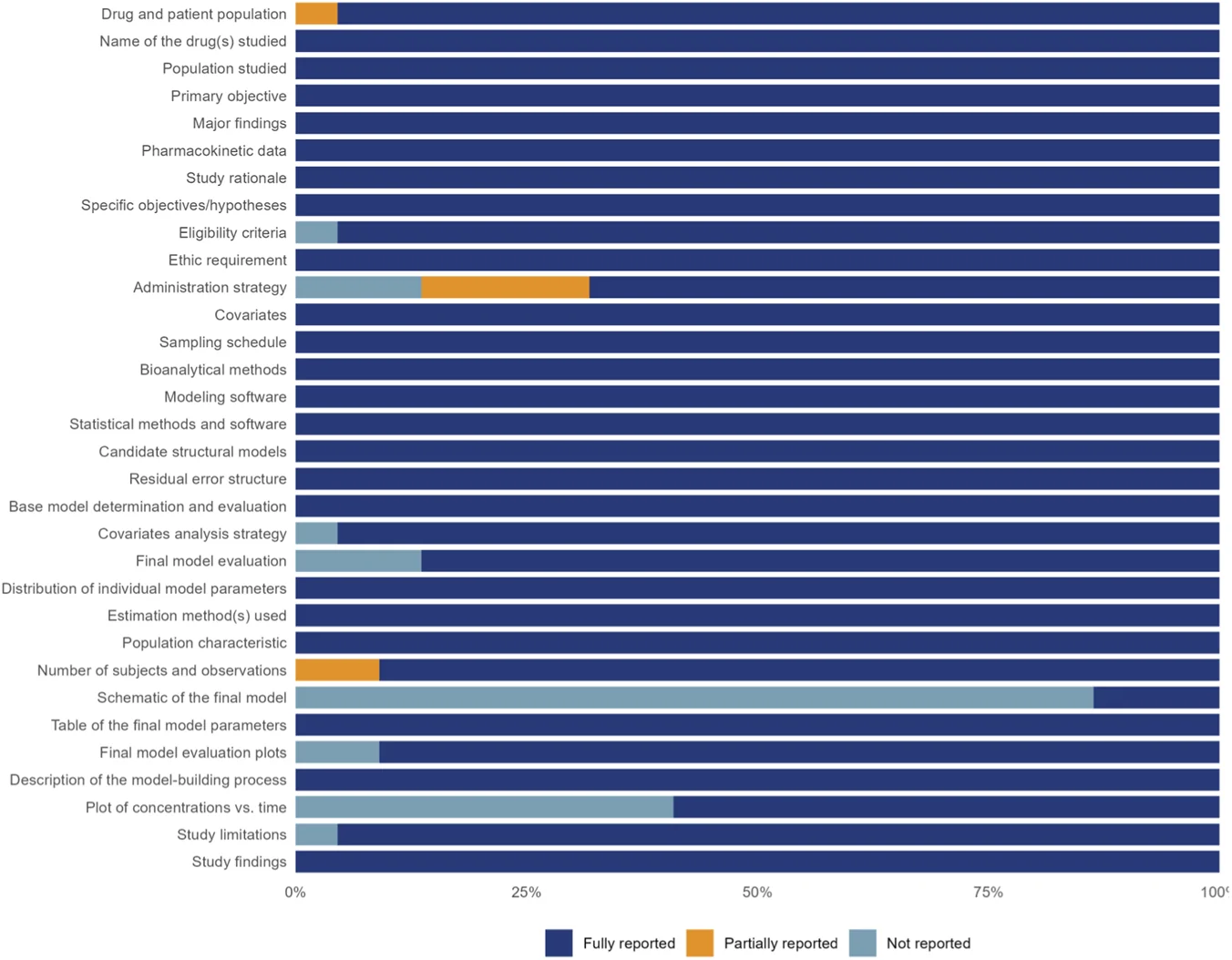
Methodological and reporting quality assessment of included PMB popPK studies. The methodological and reporting quality of the included polymyxin B population pharmacokinetic studies was evaluated using a predefined checklist comprising five domains and 32 items. Each item was scored as fully reported, partially reported, or not reported, and an overall compliance percentage was calculated for each study.

Methodological quality assessment findings are shown in Figure 1 and Supplementary Table S2. Across the included popPK studies, the median checklist adherence was 93.8%, ranging from 89.1% to 100%. The most frequently unreported item was a graphical schematic of the final model, which was absent in 19 of the 22 studies (86.4%), followed by concentration–time plots, which were absent in 9 studies (40.9%). Overall, the included studies demonstrated acceptable methodological and reporting quality. However, publication bias was not formally assessed in this study; therefore, the possibility of publication bias cannot be excluded.

### 3.2 Literature characteristics

#### 3.2.1 Basic characteristics

Across studies published from 2018 through 2025, 1,246 patients were analyzed, with ages spanning 18–104 years. Most cohorts consisted of patients treated for multidrug-resistant bacterial infections, commonly involving respiratory tract infection, sepsis, or bloodstream infection. Continuous renal replacement therapy (CRRT) and extracorporeal membrane oxygenation (ECMO) were represented in selected cohorts, although these clinically complex subgroups were unevenly covered by the published models.

In most included studies, PMB concentrations were quantified as the combined concentrations of PMB1 and PMB2, which is consistent with the Chinese consensus guidelines for PMB therapeutic drug monitoring (TDM) (Liu et al., 2023a), where PMB concentrations are calculated and reported after determining polymyxins B1 and B2 by LC-MS/MS. Four studies with relatively limited reporting detail reported total PMB concentrations without clearly specifying the component-based calculation. In addition, one study defined PMB concentration as the sum of PMB1, PMB2, and PMB1-Ile, whereas another calculated PMB concentration as the sum of PMB1, PMB2, PMB3, and isoleucine-PMB1.

Plasma PMB concentrations were measured using liquid chromatography-mass spectrometry methods. Across the included studies, the reported assay lower limits of quantification varied between 0.03 and 0.469 mg/L. Detailed study characteristics are presented in Table 1, while study-specific population definitions and eligibility criteria, including how critical illness was operationalized, are provided in Supplementary Table S3.

**TABLE 1:** Characteristics of included PMB popPK studies.

Model ID/Study (year)	Type of study	Country	Subjects	No. of subjects (M/F)	Age (years) Mean ± SD or median [range/IQR]	Weight (kg) Mean ± SD or median [range/IQR]	No. of observations	Sampling design	Dosage regimen	Route of administration	Infusion duration	Bioanalytical method (LLOQ/LLOD, mg/L)
Model 1: Yang J. (2025) Yang et al. (2025a)	Prospective	China	Severe burn patients	53 (48/5)	48.3 ± 14.08	70 [64.25–75]	118	15 min (predose) and 15 min (postdose)	maintenance dose 1.2 ± 0.35 mg/kg; loading dose in 19/53 (35.85%) patients	IV infusion	NR	UPLC-MS/MS [0.2(PMB1),0.05(PMB2)]
Model 2: Li X. Y. (2025) Li et al. (2025)	Prospective	China	Patients with pneumonia	76 (59/17)	66.0 [55.0–76.3]	65.0 [54.38–70.0]	344 (156 BAL fluid samplesand 188 plasma samples)	0 h (predose) and 1, 2, 4, 6, 8, and 12 h (postdose)	intravenous1.82 (1.54–2.17) mg/kg/day, nebulization 0.77 (0.76–0.95) mg/kg/day	IV infusion and/or nebulized administration	IV infusion: approximately 1 h; nebulization: 15 min/session	LC-MS/MS Plasma [0.156(PMB1),0.0156(PMB2)] BAL [0.1(PMB1),0.01 (PMB2)]
Model 3: Yang J. (2025) Yang et al. (2025b)	Prospective	China	critically ill patients	95(Modeling60/20; Validation 12/3)	Modeling60 (47–74); Validation 66 ± 5.26	Modeling63 (55–74); Validation 69.54 ± 3.25	214	Steady-state trough and peak concentrations (Ctrough,ss and Cpeak,ss)	Loading dose: 78/80 (97.5%) in modeling set and 12/15 (80.0%) in validation set received a loading dose; maintenance dose: 1.31 ± 0.25 mg/kg (modeling set) and 1.08 ± 0.07 mg/kg (validation set)	IV infusion; concomitant nebulization in some patients	NR	UPLC-MS/MS [0.2(PMB1),0.05 (PMB2)]
Model 4: Wang P. (2024) Wang et al. (2024)	Prospective	China	Patients with sepsis	83 (67/16)	52.0 [18.0–94.0]	65.0 [45.0–110.0]	466	0 h (predose) and at 1, 2, 3, 4 h, and 8 h (postdose)	Daily maintenance dose 150.0 (60.0–200.0) mg; Loading dose 150.0 (60.0–200.0)mg; Daily maintenance dose 2.05 (1.18–3.08) mg/kg/day	IV infusion	1.0 (0.5–2.0) h	UPLC-MS/MS [0.2(PMB1),0.05 (PMB2)]
Model 5: Li X. (2023) Li et al. (2023)	Retrospective	China	Patients with liver dysfunction	138 (106/32)	58.0 [49.0–72.3]	60.0 [55.0–67.0]	401	0 h, collected 30 min before administration; 1, 2, 4, 8, 12 h after start of infusion	1.81 (1.54–2.31) mg/kg/day	IV infusion	approximately 1 h	LC-MS/MS [0.156(PMB1),0.0156(PMB2)]
Model 6: Pi M. (2023) Pi et al. (2023)	Prospective	China	critically ill patients	30 (24/6)	55 [21–82]	63.5 [40–90]	212	0 h (predose) and at 0.1, 0.5,1,2,4,8 h and 12 h (postdose)	loading dose 150 (100–200) mg and maintenance dose 75 ( 50–100) mg q12 h	IV infusion	NR	HPLC-MS [0.1 (PMB1), 0.05 (PMB2)]
Model 7: Yu Z. (2022) Yu et al. (2022)	Prospective	China	patients with bloodstream infection	9 (7/2)	68 [63–73]	60 [55–65]	NR	Immediately before and after loading dose; immediately before second dose; then predose and 0.5, 1, 2, 3, 4, 5, 6, 7, 8, 10, and 12 h after start of infusion at the 5th-7th dose; optional 24 h sample after last infusion	loading dose 2.5 mg/kg, maintenance dose 1.25 mg/kg q12 h	IV infusion	Loading dose: 3 h; maintenance dose: 2 h	LC-MS/MS [0.05(PMB1),0.01(PMB2)]
Model 8: Wang P. (2022) Wang et al. (2022a)	Prospective	China	elderly patients	23 (20/3)	73.0 [65.0–94.0]	70.0 [50.0–83.0]	142	0 h (predose) and at 0,1,2,4,8 h (postdose)	Daily dose/weight 1.7 (1.2–2.6)mg/kg/day	IV infusion	NR	UPLC-MS/MS [0.2(PMB1),0.05(PMB2)]
Model 9: Cai X. (2022) Cai et al. (2022)	Prospective	China	lung transplant recipients	34 (25/9)	56 ± 12.76	52.15 ± 10.00	164	0.5 h (predose) and at 1,2,6 h (postdose)	intravenous daily dose 100 [100, 150] mg; aerosol inhalation 50 [50, 50] mg	IV infusion and aerosol inhalation	IV infusion: 1 h; aerosol inhalation: NR	LC-MS/MS [0.1 (PMB)]
Model 10: Luo X. (2022) Luo et al. (2022)	Prospective	China	patients with/without continuous renal replacement therapy	63 (42/21)	Modeling group 65 [27–93]; Validation group 61 [32–104]	Modeling group 60 [41–91]; Validation group 61 [48–80]	189	0 h (predose) and at 0.6 h (postdose)	Modeling group:loading dose 1.73 (1.25–2.73) mg/kg; maintenance dose2.22 (1.52–3.00)mg/kg/day; Validation group:loading dose 1.66 (1.25–2.24)mg/kg; maintenance dose 2.13 (1.25–2.94) mg/kg/day	IV infusion	NR	HPLC-MS/MS [0.07(PMB1),0.03(PMB2)]
Model 11: Wang P. (2022) Wang et al. (2022b)	Prospective	China	critically ill patients receiving CVVH	53 (29/24)	On CVVH 53.53 ± 17.95; off CVVH 54.37 ± 16.37	On CVVH 66.94 ± 15.31; off CVVH 63.99 ± 12.96	405	0 h (predose) and 1,2,4,6,8 h (postdose)	daily dose on CVVH 1.94 ± 0.53 mg/kg/day; daily dose off CVVH 2.00 ± 0.60 mg/kg/day	IV infusion	≥1 h	UPLC-MS/MS [0.2(PMB1),0.05(PMB2)]
Model 12: Wang P. (2021) Wang et al. (2021a)	Retrospective	China	obese patients	26 (17/9)	52 [18–83]	90 [75–125]	142	Rich sampling: 0, 1, 1.5, 2,4, 6, 8 h; TDM sampling: C0 and C2	1.65 (0.92–2.70)mg/kg/day	IV infusion	≥1 h	HPLC-MS [0.2(PMB1),0.05(PMB2)]
Model 13: Li Y. (2021) Li et al. (2021)	Prospective	China	Renal Transplant Patients	50 (32/18)	43.5 [18–66]	57.8 ± 12.4	151	0.5 h (predose) and 0,0.5,1,2,4,6.8 h (postdose)	50 mg q12 h (20 patients); 40 mg q 12 h (22 patients); Other doses (8 patients)	IV infusion	1–2 h	HPLC-MS/MS [PMB1 and PMB2; 0.03]
Model 14: Wang P. (2021) Wang et al. (2021b)	Retrospective	China	patients with and without renal insufficiency	70 (58/12)	normal renal:47.3 ± 17.7; renal insufficiency:54.2 ± 17.5	normal renal:68.6 ± 11.6; renal insufficiency:66.9 ± 11.1	462	0 h (predose) and 0–1,2–4,6–10 h (postdose)	2.0 ± 0.5 mg/kg/day	IV infusion	≥1 h	LC-MS/MS [0.2(PMB1),0.05(PMB2)]
Model 15: Yu X-B. (2021) Yu et al. (2021)	Retrospective	China	adult patients with various renal functions	32 (26/6)	63.63 ± 12.92	61.73 ± 11.77	112	Cmin: 30 min before next dose; Cmax: 30 min after end of infusion; urine samples collected over a 0–12 h dosing interval in 4 patients	total daily dose 136.6 ± 25.48 mg/day	IV infusion	NR	LC-MS/MS [0.1(PMB)]
Model 16: Wang P. (2020) Wang et al. (2020)	Prospective	China	patients with MDR Gramnegative bacterial infections	46 (39/7)	46 [18–94]	70 [45–98]	331	0 h (predose) and 0.5,1,1.5,2,4,6,8 h (postdose)	daily dose 1.91 [1.18–3.33] mg/kg/day and 100/150/200 mg in 25/15/8 of patients	IV infusion	0.5, 1, or 2 h	LC-MS/MS [0.2(PMB1),0.05(PMB2)]
Model 17: Xu M. (2024) Xu et al. (2024)	Prospective	China	ECMO patients	11 (8/3)	56 [48–64]	60 [57.05–66.00]	132	1, 1.5, 2, 4, 8, and 12 h after start of infusion; pre- and post-oxygenator samples	loading dose 200–300mg, 100–150 mg/day (2.5–3 mg/kg/day)	IV infusion	1 h	LC-MS/MS [0.2(PMB1),0.05(PMB2)]
Model 18: Ye Q. (2022) Ye et al. (2022)	Prospective	China	critically ill patients with or without ECMO	44 (33/11)	64.1 ± 15.9	65.3 ± 16.5	342	0 h (predose) and 0.25,0.5,1,2, 4,8,12 h (postdose)	100–200 mg/day (1.03–2.86 mg/kg/day)	IV infusion	1 h	UPLC-MS/MS [0.469 (total PMB; PMB1 + PMB2 + PMB3 + isoleucine-PMB1)]
Model 19: Liu X. (2021) Liu et al. (2021)	Prospective	China	healthy subjects	20 (10/10)	19–45	NR	285	0.75 mg/kg group,0 h (predose)and 0.5,1,1.25,1.5,2,4,6,8,10,12,16,24,36 and 48 h (postdose); 1.5 mg/kg group,0 h (predose) and 0.75,1.5,1.75,2,3,4,6,8,10,12,16,24,36 and 48 h (postdose)	The first group 0.75 mg/kg; the second group1.5 mg/kg	IV infusion	1 h; 1.5 h	LC-MS/MS [0.05(PMB1),0.011(PMB2)]
Model 20: Hanafin P. (2023) Hanafin et al. (2023)	Prospective	United States, Singapore, Brazil	hospitalized patients, including those on RRT	142 (91/51)	65 [19–94]	71 [32.5–130]	681	pre-dose; 0.5, 1, 2, and 6 h after completion of infusion; 8–12 h for once-daily dosing; approximately 1 h before next infusion	2.56 (1.33–6.00)mg/kg/day	IV infusion	1–6 h	LC-MS/MS [0.05(PMB)]
Model 21: Tang T. (2023) Tang et al. (2023)	Prospective	China	CRO pneumonia in critically ill patients	105 (76/29)	65 [55–76]	55 [50–65]	295	0 h (predose) and 0,1,2,4,6,8,10 h (postdose)	loading dose:2.1 (1.9–2.3)mg/kg; total dose/body weight 26.0 (18.7–38.2) mg/kg; daily dose 150 (100–150) mg; 2.3 (2.0–2.9) mg/kg/day	IV infusion, with concomitant nebulization in some patients	IV: ≥1 h; nebulization: NR	HPLC-MS/MS [LLOD 0.03 mg/L]
Model 22: Kubin C. (2018) Kubin et al. (2018)	Retrospective	United States	hospitalized patients	43 (30/13)	58 [39–69]	78 [59–95]	134	Troughs within 60 min before dose; peaks 15–60 min after end of infusion	2.8 (1.5–3.0)mg/kg/day	IV infusion	1h; could be lengthened as needed	HPLC-MS [0.1(PMB)]

Abbreviations: PMB, polymyxin B; popPK, population pharmacokinetic; M/F, male/female; SD, standard deviation; IQR, interquartile range; NR, not reported; IV, intravenous; BAL, bronchoalveolar lavage; MDR, multidrug-resistant; CRO, carbapenem-resistant organism; ECMO, extracorporeal membrane oxygenation; CVVH, continuous venovenous hemofiltration; RRT, renal replacement therapy; TDM, therapeutic drug monitoring; q12h, every 12 h; C0, predose concentration; C2, 2-h postdose concentration; LLOQ, lower limit of quantification; LLOD, lower limit of detection; LC-MS/MS, liquid chromatography-tandem mass spectrometry; UPLC-MS/MS, ultra-performance liquid chromatography-tandem mass spectrometry; HPLC-MS, high-performance liquid chromatography-mass spectrometry; HPLC-MS/MS, high-performance liquid chromatography-tandem mass spectrometry; PMB1, polymyxin B1; PMB2, polymyxin B2; PMB3, polymyxin B3; B1-Ile, isoleucine-polymyxin B1.

Sampling frequency = Sample counts/Number of subjects.

#### 3.2.2 Population pharmacokinetic characteristics

Model development was performed using NONMEM (n = 10), Phoenix NLME (n = 11) or Monolix (n = 1). Among estimation approaches, first order conditional estimation with interaction was used most frequently. PMB pharmacokinetics was generally characterized by a two compartment structure with first order absorption and elimination. Model evaluation commonly relied on visual predictive checks (VPCs), bootstrap procedures, and goodness of fit (GOF) diagnostics. Detailed model characteristics and pharmacokinetic parameter estimates are summarized in Table 2.

**TABLE 2:** Population pharmacokinetic parameters of retrieved studies.

Model ID/Study (year)	Software/Algorithm	Structural model	Fixed effect parameters		Covariate effect	Interindividual variability	Residual variability (Prop: %; Add: Mg/L; stdev0 as reported)	Internal validation	External validation (N = number of samples)	Model application
Model 1: Yang J. (2025) Yang et al. (2025a)	NONMEM 7.5.1 (FOCE-I)	1 CMT with FO elimination	CL	= 2.50×exp [(-0.0207) × (BUN-9.5)] L/h	BUN = 9.50 (6.40–13.01)	34.1%	Expo:0.34	Bootstrap VPC	NR	Dose recommendations based on AUC_ss,24h_ (and AUC_ss,24h_/MIC PTA)with BUN-guided individualization
			Vd	= 19.1 (L)		-				
Model 2: Li X. Y. (2025) Li et al. (2025)	NONMEM 7.5 (FOCE-I)	Coupled 2 CMT models for plasma and ELF with FO elimination (IV to plasma, nebulized to ELF)	CL1	= 1.39L/h	AGE on V3 = 1.28; ALB on CL3 = −0.893	56%	Prop: 43%	Bootstrap VPC GOF	NR	Dose recommendations based on ELF PTA (fAUC/MIC) and plasma AUC (toxicity threshold 100 mg⋅h/L)
			V1	= 27.6 L		31%				
			CL2	= 13.11 L/h		​				
			V2	= 17.1 L		​				
			CL3	= 0.186 L/h		45%				
			V3	= 0.896 L		53%				
			CL4	= 3.44 L/h		​				
			V4	= 4.35 L		​				
			Q	= 0.03L/h		​				
			F	= 0.557		​				
Model 3: Yang J. (2025) Yang et al. (2025b)	NONMEM 7.5.1 (FOCE-I)	1 CMT with FO elimination	CL	CL = 2.03 × (CrCL/75.99)^0.26^ × (PLT/163.5)^−0.14^ L/h	CrCL = 75.99 (38.46–130.58) PLT = 163.50 (84.5–266.25)	38.5%	Prop: 30% Add:0.21	Bootstrap VPC GOF NPDE	N = 30	Dose Recommendations Based on Cmax and AUC0-24
			Vd	= 18 L		​				
Model 4: Wang P. (2024) Wang et al. (2024)	Phoenix NLME 8.3 (FOCE-ELS)	2-CMT with linear elimination	Vc	= 10.05×(DFI/4.23​)^0.27^ L	DFI = 4.2 (1.3–8.4) L CrCL = 87.5 (17.3–309.7)	18.26%	stdev0 = 0.1034	Bootstrap VPC GOF	NR	Dose recommendations based on Cmax and AUC0-24
			Vp	= 18.78 L		29.87%				
			CL	= 1.97×(CrCL/87.46)^0.39^×(DFI/4.23)^0.28^L/h		16.06%				
			Q	= 2.99 L/h		47.38%				
Model 5: Li X. (2023) Li et al. (2023)	Phoenix NLME 8.1(FOCE-ELS)	1 CMT with FO elimination	CL	= 2.43×(CrCL/52.9) ^0.22^×exp [0.15×I(CP = B)] × exp [0.16×I(CP = C)] L/h	CrCL = 52.1 (34.5–96.1)	19%	Prop: 26%	Bootstrap pc-VPC GOF AIC	NR	Dose recommendations based on AUC0-24 and Cmax, and probabilities of target attainment (PTA) for different dosing regimens
			V	= 23.11 L		10%				
Model 6: Pi M. (2023) Pi et al. (2023)	Phoenix NLME8.1 (FOCE-ELS)	2-CMT with linear elimination	CL	= 1.672× (WT/63.817)^2.031^ × exp [1.114 × I(CRRT)] L/h	WT = 63.5 (40–90)	28.2%	-	Bootstrap VPC GOF AIC BIC BA	NR	Dose recommendations based on C0.5h–8 h sampling, body weight, and CRRT
			Q	= 7.009 × (WT/63.817)^3.408^ L/h		35.6%				
			V	= 7.857 L		38.7%				
			V2	= 12.668 L		64.5%				
Model 7: Yu Z. (2022) Yu et al. (2022)	NONMEM 7.4 (FOCE-I)	2 CMT with linear elimination	CL	= 1.60 L/h	θDIS on V1 = 0.371; θAGE on V2 = 1.67	18.2%	Prop: 11.8%	Bootstrap VPC GOF	NR	Probabilities of target attainment: AUC_ss,24h_/MIC of ≥54.4
			Q	= 3.45 L/h		fixed/0%				
			V1	= 38.6 L		20.0%				
			V2	= 7.13×(AGE/33)^1.67^ L		27.2%				
Model 8: Wang P. (2022) Wang et al. (2022a)	Phoenix NLME 8.3(FOCE)	2-CMT with linear elimination	V	= 8.13×(Albumin/30.3) ^1.18 L	ALB = 30.3 (18.3–43.5)	6%	stdev0 = 0.0621	Bootstrap pc-VPC GOF	NR	Dose recommendations are based on AUC/MIC and AUC_ss,24h_ to optimize efficacy while minimizing nephrotoxicity in elderly patients with multi-drug-resistant Gram-negative bacterial infections
			V2	= 19.67 L		25%				
			CL	= 1.87 L/h		12%				
			Q	= 6.45 L/h		38%				
Model 9: Cai X. (2022) Cai et al. (2022)	NONMEM 7.4 (FOCE-I)	1 CMT with FO elimination	CL	= 1.72×(CrCL/78.49)^0.681^L/h	CrCL = 80.81 ± 29.97	32.6%	Prop: 37.9%	Bootstrap pc-VPC GOF NPDE	NR	Dose recommendations based on Cmax and AUC0-24
			V	= 14.4 L		40.6%				
Model 10: Luo X. (2022) Luo et al. (2022)	Pirana 2.9.0	2-CMT with linear elimination	CL	= [(1-CRRT)×1.5×(CLCR/83.69)^0.506^+CRRT×1.95] × exp (0.0447×(SOFA - 6)) L/h	SOFA = 6 (0–22) CLCR = 83.69 (13.48–197.84)	5.98%	Prop:5.89%	Bootstrap GOF	N = 42	Dose recommendations based on Cmax and AUC0-24
			V1	= 11.7 L		​				
			Q	= 1.34 L/h		​				
			V2	= 17.9 L		85.4%				
Model 11: Wang P. (2022) Wang et al. (2022b)	Phoenix NLME 7.0(FOCE-ELS)	2-CMT with linear elimination	V	= 15.0 L	dV2dCRRT = 2.53; dCLdCRRT = 0.841; dQdCRRT = 1.36	13%	stdev0 = 0.1303	Bootstrap VPC GOF	NR	Dose recommendations based on Cmax and AUC0-24
			V2	= 6.54 L		138%				
			CL	= 1.95 L/h		17%				
			Q	= 2.28 L/h		51%				
Model 12: Wang P. (2021) Wang et al. (2021a)	Phoenix NLME 7.0(FOCE-ELS)	2-CMT with linear elimination	V	= 11.24 L	-	​	stdev0 = 0.24	Bootstrap pc-VPC GOF	NR	Dose recommendations based on Cmax and AUC0-24
			V2	= 39.70 L		100%				
			CL	= 2.86 L/h		17%				
			Q	= 7.36 L/h		43%				
Model 13: Li Y. (2021) Li et al. (2021)	Phoenix NLME 8.1(FOCE-ELS)	1 CMT with FO elimination	CL	= 1.18×(CrCL/22.2)^0.14^ L/h	CrCL = 22.2 (4.29–90.7)	6%	Prop:17%	Bootstrap Pc-VPC GOF	NR	Dose recommendations based on Cmax and AUC0-24
			V	= 12.09 L		4%				
Model 14: Wang P. (2021) Wang et al. (2021b)	Phoenix NLME 7.0(FOCE-ELS)	2CMT with FO elimination	V	= 6.87 L	CrCL = 123.3 (81.6–315.2)	78%	stdev0 = 0.13	Bootstrap VPC GOF	NR	Dose recommendations were guided primarily by model-derived AUC_ss,24h_, with Css,min used as a practical surrogate exposure metric
			V2	= 11.97 L		32%				
			CL	= 2.19 L/h		22%				
			Q	= 13.83 L/h		68%				
			V	= 6.98 L	CrCL = 42.0 (15.6–77.6)	38%	stdev0 = 0.10			
			V2	= 10.57 L		74%				
			CL	= 1.58 L/h		26%				
			Q	= 10.28 L/h		-				
Model 15: Yu X-B. (2021) Yu et al. (2021)	NONMEM 7.4 (FOCE-I)	1 CMT with FO elimination	CL	= 1.59×(CrCL/80)^0.408^ L/h	CrCL = 86.58 ± 53.00 mL/min; model normalized to CrCL/80	13%	Prop:40.5%	pc-VPC GOF NPDE	NR	Dose recommendations via Monte Carlo simulation-based PTA for AUC__ss,24h_ and fAUC/MIC ≥ 20, with CrCL-stratified regimens
			Vi	= 20.5 L		​				
Model 16: Wang P. (2020) Wang et al. (2020)	Phoenix NLME 7.0(FOCE)	2CMT with FO elimination	V	= 6.218 L	CrCL = 89.3 (15.6–315.2)	31.8%	stdev0 = 0.110	Bootstrap Pc-VPC GOF	NR	Dose recommendations to achieve target exposure (AUCss,24 50–100 mg h/L; Css,avg 2–4 mg/L),plus LSS models to estimate AUC
			V2	= 11.922 L		69.0%				
			CL	= 1.786×(CrCL/105.9)^0.362 L/h		20.8%				
			Q	= 13.518 L/h		150.8%				
Model 17: Xu M. (2024) Xu et al. (2024)	Phoenix NLME 8.1(FOCE)	2CMT with FO elimination	CL	= 3.75 L/h	dVdCRRT0 = −0.525; dCLdspeedECMO = −0.321	3.8%	stdev0 = 0.361	Bootstrap Pc-VPC GOF	NR	Dose recommendations based on Css and AUC0-24
			V	= 20.41 L		6.2%				
			Vp	= 9.86 L		​				
			Q	= 3.82 L/h		​				
Model 18: Ye Q. (2022) Ye et al. (2022)	NONMEM 7.2	2CMT with FO elimination	CL	= 1.27×(CLCR/43.3)^1.22^ L/h	CrCL = 43.3 (32.5–66.8)	15.1%	Add:0.10	Bootstrap Pc-VPC GOF	NR	Dose recommendations based on ssAUC0-24 (target ≥50 mg h/L) and AUC/MIC PTA
			Vc	= 8.85 L		15.4%				
			Vp	= 10.4 L		​				
			Q	= 5.42 L/h		33.2%				
Model 19: Liu X. (2021) Liu et al. (2021)	NONMEM 7.4 (FOCE-I)	3CMT with linear central clearance	CL	= 0.027 L/h/kg	θSex on V3 = 0.73	11%	Prop:5.15%	Bootstrap VPC GOF	NR	Dose optimization based on exposure (AUC) to meet the PK/PD target (fAUC/MIC), with safety-driven upper-limit considerations and support for TDM
			V1	= 0.071 L/kg		21%				
			V2	= 0.061 L/kg		17%				
			V3	= 0.045 L/kg		14%				
			Q2	= 0.14 L/h/kg		​				
			Q3	= 0.0064×[1 + 0.051×(Age−28.5)] L/h/kg		​				
Model 20: Hanafin P. (2023) Hanafin et al. (2023)	NONMEM 7.4.3 (FOCE)	2CMT with FO elimination; CVVHDF and CrCL were included as covariates on CL, and BW as a covariate on Vc	CL	= [(1-I(CVVHDF))×1.19×(CLCR/55.2)^0.242^ + I(CVVHDF) ×1.44] L/h	​	20.88%	add:0.593 Prop:7.67%	Bootstrap Pc-VPC GOF OFV	NR	Dose recommendations based on simulated exposure (Cmax, AUC0-24) and PTA (fAUC/MIC target)
			V1	= 11.8× (BW/71)^1^ L		39.31%				
			Q	= 1.44 L/h		​				
			V2	= 7.9 L		​				
Model 21: Tang T. (2023) Tang et al. (2023)	Phoenix NLME 8.1(FOCE-ELS)	2-CMT with linear elimination	CL	= 1.56 L/h	-	CL: 39.4%; Corr (CL,Vc): 100%	Prop:27.86%	Bootstrap Pc-VPC GOF OFV	NR	Monte Carlo simulation based on AUC_ss,24h_/MIC; 75 or 100 mg q12 h for MIC ≤0.5 mg/L
			CLd	= 2.41 L/h		​				
			Vc	= 12.5 L		28.6%				
			Vp	= 29.9 L		​				
Model 22: Kubin C. (2018) Kubin et al. (2018)	Monolix 2016R1 (NLME)	1 CMT with FO elimination	CLpop	= 2.37 L/h	-	37.7%	add:0.00693 Prop:23.3%	GOF NPDE PWRES/IWRES	NR	Dose recommendations via a free PK simulator
			Vpop	= 34.4 L		15.7%				

Abbreviations: Add, additive residual error; AIC, akaike information criterion; ALB, albumin; AUC, area under the concentration-time curve; AUC0-24, area under the concentration-time curve from 0 to 24 h; AUC/MIC, AUC-to-minimum inhibitory concentration ratio; BA, bioavailability; BIC, bayesian information criterion; BUN, blood urea nitrogen; BW, body weight; CL, clearance; CLCR, or CrCL, creatinine clearance; CMT, compartment; CP, Child-Pugh class; CRRT, continuous renal replacement therapy; CVVHDF, continuous venovenous hemodiafiltration; DFI, daily fluid intake; ELF, epithelial lining fluid; FO, first-order; FOCE, first-order conditional estimation; FOCE-I, first-order conditional estimation with interaction; FOCE-ELS, first-order conditional estimation-extended least squares; GOF, goodness of fit; IIV, interindividual variability; IV, intravenous; LSS, limited sampling strategy; MIC, minimum inhibitory concentration; NLME, nonlinear mixed-effects modeling; NONMEM, nonlinear mixed-effects modeling software; NPDE, normalized prediction distribution error; NR, not reported; OFV, objective function value; pc-VPC, prediction-corrected visual predictive check; PK, pharmacokinetic; PK/PD, pharmacokinetic/pharmacodynamic; PLT, platelet count; Prop, proportional residual error; PTA, probability of target attainment; PWRES/IWRES, population/individual weighted residuals; Q, intercompartmental clearance; SOFA, sequential organ failure assessment; TDM, therapeutic drug monitoring; V, V1, V2, V3, V4, vc, and Vp, volumes of distribution or compartment volumes as defined in each source model; VPC, visual predictive check; WT, body weight.

Across all included studies, interindividual variability (IIV) was modeled using an exponential error structure. Most models reported IIV estimates for at least one pharmacokinetic parameter, most frequently clearance and volume of distribution parameters. However, the reporting format of IIV varied across studies, limiting direct quantitative comparison of variability estimates. Residual unexplained variability was most commonly modeled using a proportional error structure, which was applied in 15 studies. Additive, exponential, and combined proportional-additive error models were reported in 2, 1, and 3 studies, respectively. For studies using proportional residual error, all reported RUV estimates were below 50%, whereas additive residual error estimates ranged from 0.000693 to 0.593.

Internal model evaluation was reported for all included models, mainly through goodness of fit (GOF) diagnostics, bootstrap analysis, and visual predictive checks (VPCs). Normalized prediction distribution error (NPDE) was additionally used in four studies. External validation with independent datasets was conducted in only two studies, and both showed acceptable predictive performance. Broader use of external validation is therefore warranted to better establish the transportability and predictive reliability of population pharmacokinetic models.

### 3.3 Covariates of inclusion

**FIGURE 2:**
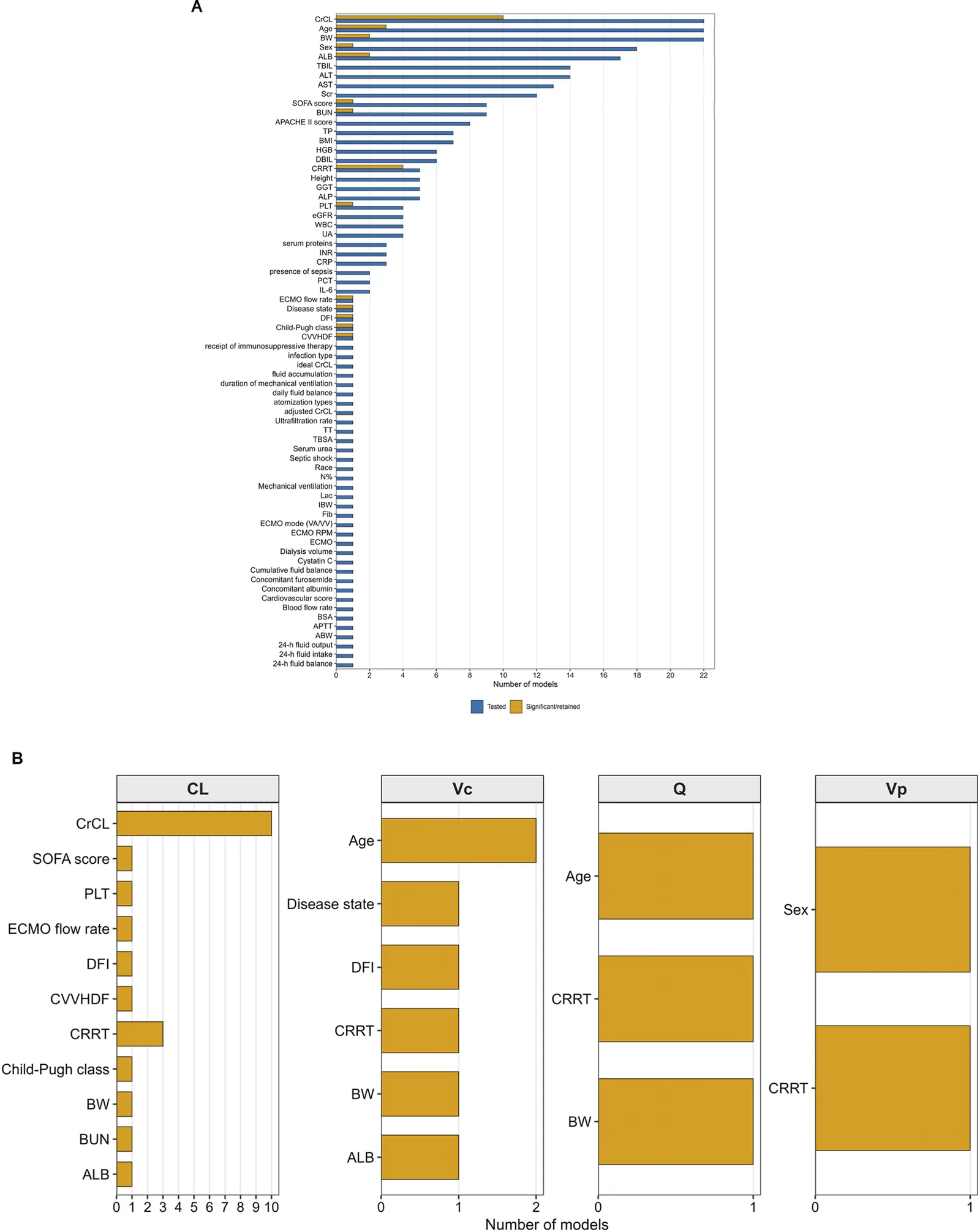
Covariates evaluated and retained in the included polymyxin B population pharmacokinetic models. **(A)** Overall covariate assessment. Blue bars represent the total number of models in which each covariate was tested, whereas orange bars represent the subset of models in which the covariate was retained in the final model as statistically significant. **(B)** Significant/retained covariates stratified by pharmacokinetic parameter (CL, Vc, Q, and Vp).

**FIGURE 3:**
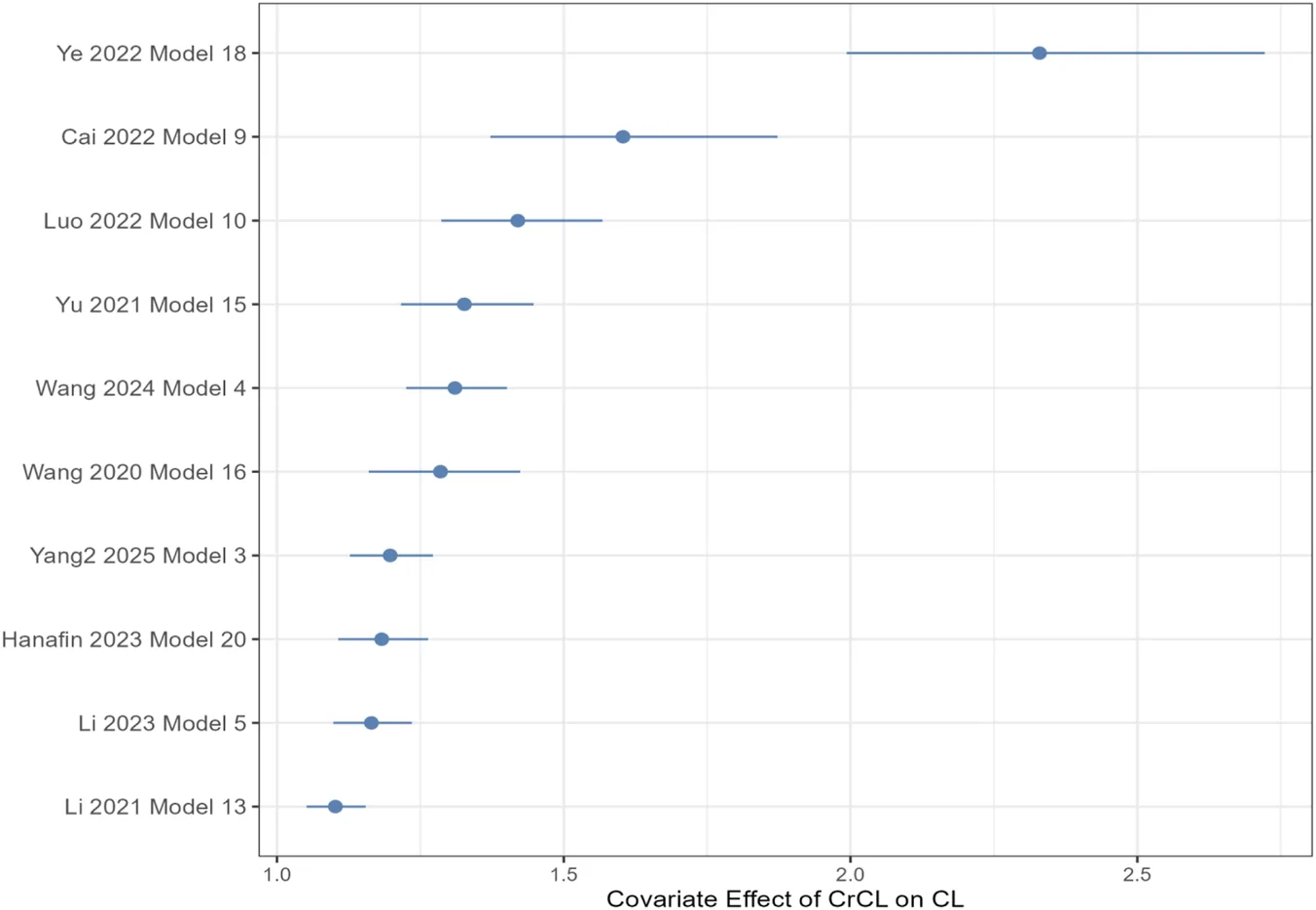
Magnitude of covariate effects on polymyxin B clearance. The relative effects of clinically relevant covariates on polymyxin B clearance were calculated using the reported covariate ranges from each included model. Covariate effects were expressed as percentages relative to the corresponding study-specific reference clearance.

Most studies assessed covariates to characterize sources of interindividual variability in PMB pharmacokinetics, with emphasis on factors considered clinically relevant. Covariate selection was commonly performed using a stepwise procedure based on forward addition followed by backward deletion. Table 3 summarizes the covariates evaluated and retained for CL and Vd, while Figure 2 shows study specific covariates tested for CL, including both significant and nonsignificant factors. Frequently examined variables included age, sex, body weight (BW), creatinine clearance (CrCL), albumin (ALB), and continuous renal replacement therapy (CRRT). Ten studies evaluated creatinine clearance estimated by the Cockcroft-Gault equation as a covariate, with higher CrCL generally corresponding to increased PMB clearance. CRRT was assessed in four studies and was likewise linked to greater clearance in the corresponding models. The magnitude of covariate effects on PMB clearance is summarized in Figure 3.

**TABLE 3:** List of tested and significant covariates in the model.

Model ID/Study (year)	Tested covariates			Covariate selection criteria		Significant covariates			
	Demographic	Laboratory tests	Others	Forward inclusion	Backward elimination	CL	Vc	Q	Vp
Model 1: Yang J. (2025) Yang et al. (2025a)	Age, BW, BMI	BUN, Scr, Cystatin C, CrCL, eGFR, ALT, AST, ALP, GGT, ALB, TBIL, WBC, HGB, PLT, INR, TT	TBSA	P < 0.05	P < 0.01	BUN	NR	NR	NR
Model 2: Li X.Y. (2025) Li et al. (2025)	Age, Sex,BW	Serum urea, CrCL,TBIL, ALB,AST,ALT, GGT,ALP,IL-6,CRP	APACHE II score, SOFA score, presence of sepsis, duration of mechanical ventilation, atomization types, receipt of immunosuppressive therapy	P < 0.05	P < 0.01	ALB	AGE	NR	NR
Model 3: Yang J. (2025) Yang et al. (2025b)	Age, BW	BUN, Scr, CrCL, eGFR, ALT, AST,TP,ALB, TBIL, HGB, WBC, PLT, IL-6, INR, APTT, Fib	APACHE II score, presence of sepsis	P < 0.05	P < 0.01	CrCL,PLT	NR	NR	NR
Model 4: Wang P. (2024) Wang et al. (2024)	Age, BW, Sex	ALB, CrCL	Septic shock, Mechanical ventilation, APACHE II score, SOFA score, DFI, daily fluid balance, Cumulative fluid balance, fluid accumulation	P < 0.05	P < 0.01	CrCL, DFI	DFI	NR	NR
Model 5: Li X. (2023) Li et al. (2023)	Age, BW, Sex, Height	TBIL, DBIL, TP, ALB, ALT, AST, GGT, ALP, INR, CrCL	Child-Pugh class	P < 0.05	P < 0.001	CrCL, CPB,CPC	NR	NR	NR
Model 6: Pi M. (2023) Pi et al. (2023)	Age, Sex, BW, Height	TBIL, DBIL, TP, ALB, BUN, Lac, Scr, CrCL	Cardiovascular score, 24-h fluid intake, 24-h fluid output, CRRT	P < 0.01	P < 0.001	BW, CRRT	NR	BW	NR
Model 7: Yu Z. (2022) Yu et al. (2022)	Age, Sex, BW, Height, BMI	CrCL, ALB, HGB, TP, TBIL, ALT, AST,Scr	SOFA score, Disease state	P < 0.05	P < 0.001	NR	Disease state, Age	NR	NR
Model 8: Wang P. (2022) Wang et al. (2022a)	Age, BW, Sex	Scr, ALT, AST, serum proteins, ALB, TBIL, CrCL	SOFA score, APACHE II score	P < 0.05	P < 0.01	NR	ALB	NR	NR
Model 9: Cai X. (2022) Cai et al. (2022)	Age, Sex, BW, Height	HGB, WBC, N%, PLT, ALT, AST, TBIL, ALB, TP, CRP, PCT, BUN, Scr, CrCL	Concomitant furosemide, Concomitant albumin	P < 0.05	P < 0.01	CrCL	NR	NR	NR
Model 10: Luo X. (2022) Luo et al. (2022)	Age, BW, Sex	ALT, AST, ALP, GGT, TBIL,TP, BUN, UA, Scr, CrCL	APACHE II score, SOFA score, CRRT	P < 0.05	P < 0.01	CRRT, CrCL, SOFA score	NR	NR	NR
Model 11: Wang P. (2022) Wang et al. (2022b)	Age, BW, Sex	ALT, AST, TBIL, BUN, Scr, UA,serum proteins, ALB, CrCL, eGFR	SOFA score, CRRT, Dialysis volume, Blood flow rate, Ultrafiltration rate	P < 0.01	P < 0.001	CRRT	NR	CRRT	CRRT
Model 12: Wang P. (2021) Wang et al. (2021a)	Age, BW, BMI, IBW, ABW, Sex	CrCL, adjusted CrCL, ideal CrCL, Scr, eGFR	SOFA score	P < 0.01	P < 0.001	NR	NR	NR	NR
Model 13: Li Y. (2021) Li et al. (2021)	Age, BW	ALT, AST, TBIL, DBIL, ALB, BUN, UA, CrCL	NR	P < 0.05	P < 0.01	CrCL	NR	NR	NR
Model 14: Wang P. (2021) Wang et al. (2021b)	Age, BW, Sex	CrCL	NR	P < 0.05	P < 0.01	NR	NR	NR	NR
Model 15: Yu X-B. (2021) Yu et al. (2021)	Age, Sex, BW	CrCL, serum albumin concentration	NR	P < 0.05	P < 0.01	CrCL	NR	NR	NR
Model 16: Wang P. (2020) Wang et al. (2020)	Age, Sex, BW	ALT, AST, GGT, ALP, BUN, Scr, UA, serum proteins, ALB, TBIL, DBIL, CrCL	NR	P < 0.05	P < 0.01	CrCL	NR	NR	NR
Model 17: Xu M. (2024) Xu et al. (2024)	Age, Sex, BW, Height, BMI, BSA	BUN, Scr, CrCL, WBC, HGB, PLT, ALT, ALB, TBIL, DBIL, PCT, CRP	APACHE II score, SOFA score, CRRT, ECMO RPM, ECMO flow rate, ECMO mode (VA/VV)	P < 0.01	P < 0.001	ECMO flow rate	CRRT	NR	NR
Model 18: Ye Q. (2022) Ye et al. (2022)	Age, Sex, BW	ALB, CrCL	24-h fluid balance, CRRT, ECMO, SOFA score, APACHE II score	P < 0.01	P < 0.001	CrCL	NR	NR	NR
Model 19: Liu X. (2021) Liu et al. (2021)	Age, Sex, BW, BMI	Scr, CrCL, ALT, AST,ALB, HGB, TP	NR	P < 0.05	P < 0.01	NR	NR	Age	Sex
Model 20: Hanafin P. (2023) Hanafin et al. (2023)	BW, BMI,Age,Sex,Race	CrCL	APACHE II score, infection type, RRT modality, especially CVVHDF	P < 0.05	P < 0.01	CrCL, CVVHDF	BW	NR	NR
Model 21: Tang T. (2023) Tang et al. (2023)	BW, Age,Sex	ALT,AST,TBIL, DBIL,ALB,CrCL	NR	P < 0.05	P < 0.001	NR	NR	NR	NR
Model 22: Kubin C. (2018) Kubin et al. (2018)	BW, Age, BMI	CrCL	NR	P < 0.05	NR	NR	NR	NR	NR

Abbreviations: ABW, adjusted body weight; ALB, albumin; ALP, alkaline phosphatase; ALT, alanine aminotransferase; APACHE II, Acute Physiology and Chronic Health Evaluation II; APTT, activated partial thromboplastin time; AST, aspartate aminotransferase; BMI, body mass index; BSA, body surface area; BUN, blood urea nitrogen; BW, body weight; CL, clearance; CPB, Child-Pugh class B; CPC, Child-Pugh class C; CrCL, creatinine clearance; CRP, C-reactive protein; CRRT, continuous renal replacement therapy; CVVHDF, continuous venovenous hemodiafiltration; DBIL, direct bilirubin; DFI, daily fluid intake; ECMO, extracorporeal membrane oxygenation; eGFR, estimated glomerular filtration rate; Fib, fibrinogen; GGT, gamma-glutamyl transferase; HGB, hemoglobin; IBW, ideal body weight; IL-6, interleukin-6; INR, international normalized ratio; Lac, lactate; N%, neutrophil percentage; NR, not reported; OFV, objective function value; PCT, procalcitonin; PLT, platelet count; Q, intercompartmental clearance; RPM, revolutions per minute; RRT, renal replacement therapy; Scr, serum creatinine; SOFA, sequential organ failure assessment; TBIL, total bilirubin; TBSA, total body surface area; TP, total protein; TT, thrombin time; UA, uric acid; VA, venoarterial; Vc, central compartment volume of distribution; Vp, peripheral compartment volume of distribution; VV, venovenous; WBC, white blood cell count.

### 3.4 Exposure comparison

**FIGURE 4:**
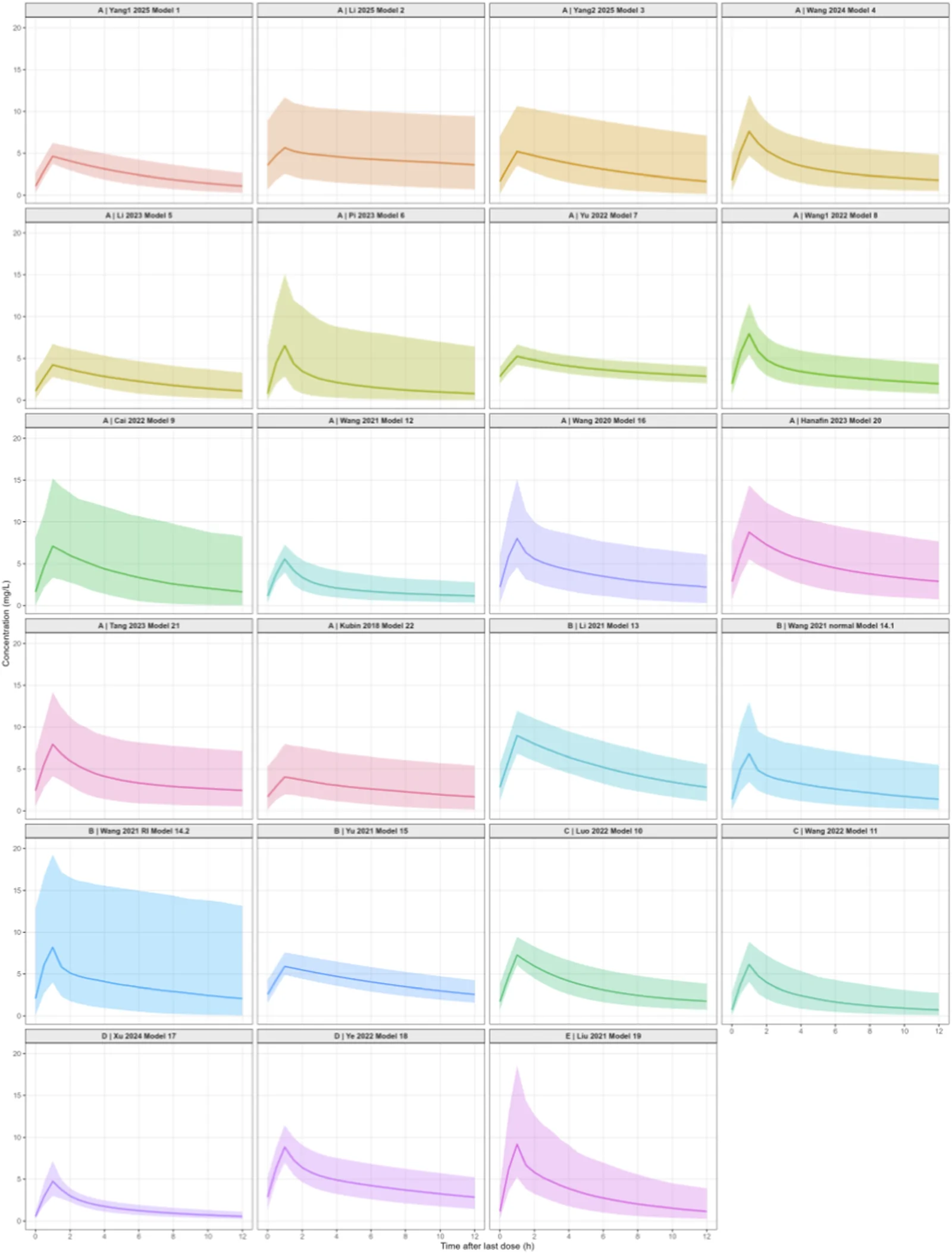
Simulated concentration-time profiles of polymyxin B based on included population pharmacokinetic models. Monte Carlo simulations were performed using the reconstructed population pharmacokinetic models in the mrgsolve framework. For each model, 1,000 virtual patients were simulated under standardized dosing conditions to generate polymyxin B concentration-time profiles and predictive distributions. The figure illustrates between-model differences in predicted exposure over time. Model IDs correspond to the unified Model IDs listed in Tables 1, 2. A, patients with various infections or critical illness; B, patients with varying renal function; C, patients receiving continuous renal replacement therapy (CRRT); D, patients receiving extracorporeal membrane oxygenation (ECMO); and E, healthy volunteers.

**FIGURE 5:**
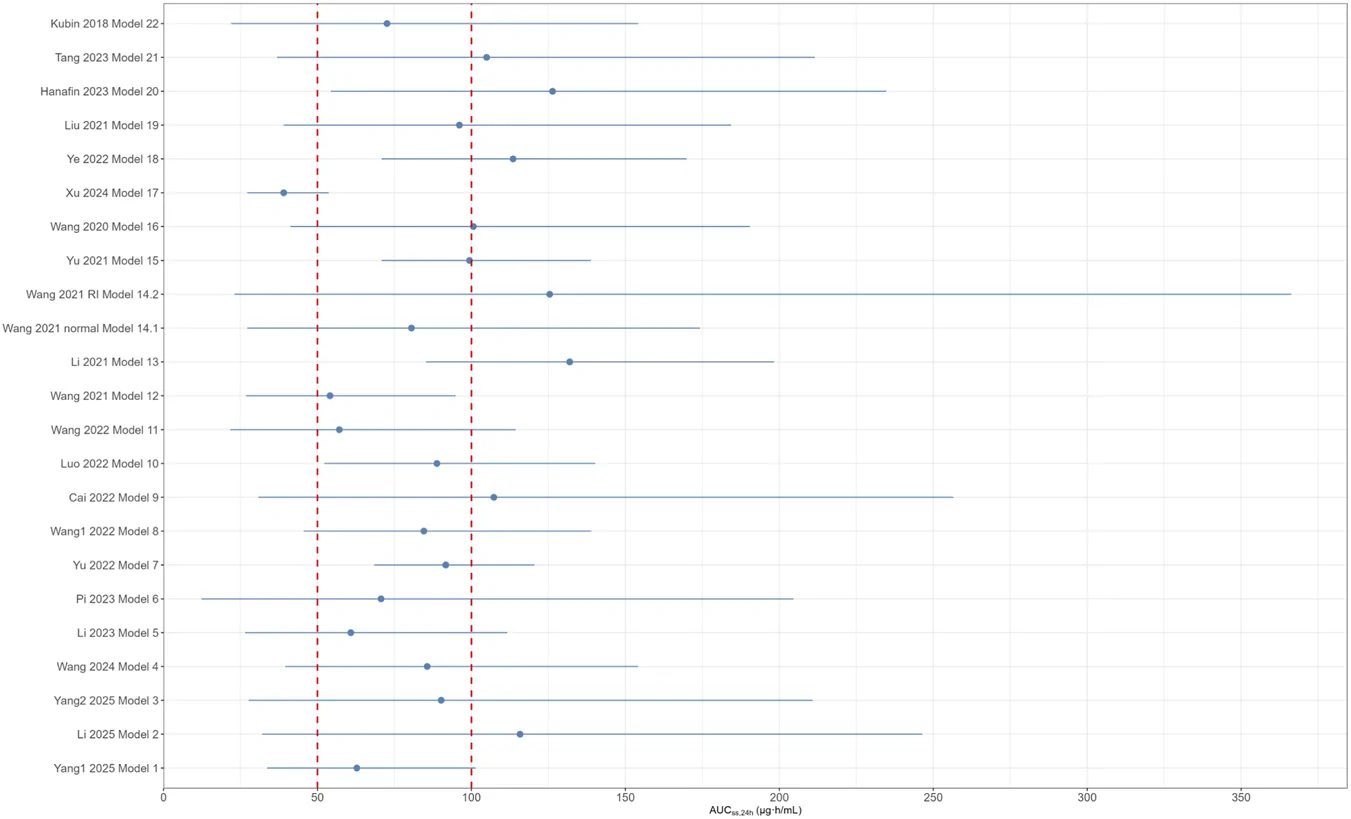
Comparison of simulated steady-state 24-h area under the concentration-time curve across polymyxin B population pharmacokinetic models. Steady-state 24-h area under the concentration-time curve values were calculated from simulated concentration-time profiles under standardized dosing conditions. The therapeutic exposure window was defined as AUC_ss,24h_ of 50–100 mg h/L, corresponding to the consensus target steady-state concentration range of approximately 2–4 mg/L. Differences across models indicate variability in predicted target attainment among clinically relevant populations. Model IDs correspond to the unified Model IDs listed in Tables 1, 2.

Figure 4 shows the simulated concentration-time curves generated from the included population pharmacokinetic models. Using these simulated profiles, AUC_ss,24h_ values were calculated for virtual patients and are summarized in Figure 5. For most study populations, a dosing regimen of 75 mg administered as a 1-h intravenous infusion yielded AUC_ss,24h_ values within the therapeutic window of 50–100 μg h/mL. In patients with renal impairment, Model 14 (Wang et al., 2021b) and Model 15 (Yu et al., 2021) indicated that exposure at the same dose was higher than that in patients with normal renal function, with some individuals exceeding the therapeutic window, thereby potentially increasing the risk of acute kidney injury. In renal transplant recipients, Model 13 (Li et al., 2021) indicated that nearly all patients exceeded the therapeutic window when receiving a 75 mg maintenance dose. Therefore, for patients with impaired renal function, clinicians should consider reduced doses to mitigate the risk of adverse events. Under the 75 mg 1-h infusion regimen, the exposure and pharmacokinetic behavior of polymyxin B were comparable across the majority of typical study populations such as Model 4 (Wang et al., 2024), Model 8 (Wang et al., 2022a), and Model 10 (Luo et al., 2022). The present study was designed to develop a population pharmacokinetic model repository for polymyxin B and to evaluate clinically relevant covariate effects that may support model-informed individualized dosing.

### 3.5 Model application

All scripts used to build the PMB model repository and its Shiny-based interface are publicly accessible at https://github.com/luobo-qc/polymyxin-b-model-library. Representative views of the application interface are shown in Supplementary Figure S2.

## 4 Discussion

The present work collated published polymyxin B popPK models and translated them into a unified mrgsolve based repository. Building on this repository, we constructed a Shiny application to support exposure prediction and individualized dosing evaluation (Tam et al., 2020; Oda et al., 2023). The repository brings together essential model-level and study-level details, covering patient demographics and clinical features, dosing schedules, sampling designs, bioanalytical methods, modeling approaches, and covariate structures. Model based simulations were then performed to compare exposure across representative study populations and to evaluate whether standard dosing regimens could achieve predefined therapeutic targets.

**FIGURE 6:**
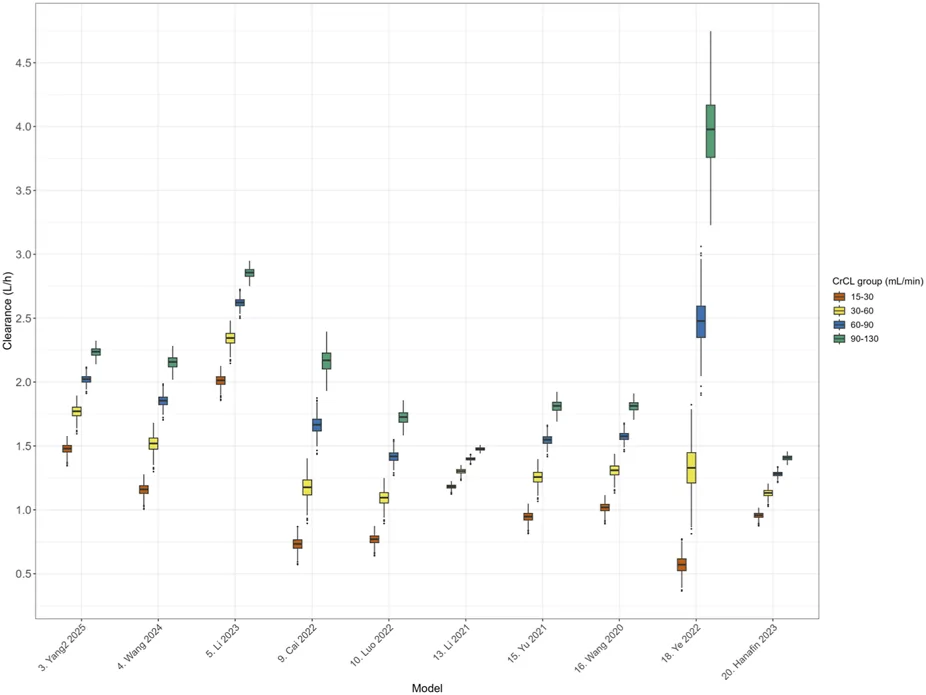
Model-based simulations of clearance (CL) in patients with varying renal function.

PMB is primarily excreted unchanged via the biliary route, with only a minor fraction eliminated renally (Zavascki et al., 2008; Sandri et al., 2013). During tubular reabsorption, PMB is taken up into renal tubular epithelial cells by transporter proteins (Lu et al., 2016), leading to substantial intracellular accumulation of the drug. Given this distinctive elimination pathway, previous studies suggested that renal function had little effect on PMB elimination, reporting either no marked difference in AUC_ss_,_24h_ between patients with renal impairment and those with normal renal function (Sandri et al., 2013) or only a minimal effect on PMB clearance (Zamri et al., 2025). Accordingly, international consensus guidelines have considered dose adjustment unnecessary for patients with renal impairment (Tsuji et al., 2019). However, among the 22 reviewed studies, renal function indices were retained as significant predictors in 10 models, affecting either PMB clearance or volume of distribution. By integrating models from multiple studies and performing Monte Carlo simulations (Supplementary Figure S3), we found that patients with renal impairment exhibit higher drug exposure than those with normal renal function when receiving the same dose (Wang et al., 2021b; Yu et al., 2021). Model-predicted CL across renal-function strata is shown in Figure 6. In addition, we observed that the estimated CL values in Chinese populations were generally lower than those reported in Western populations. We therefore recommend that users consider ethnic factors when applying the R Shiny platform for dose prediction. Considering the complexity of current clinical practice, we recommend formulating dosing strategies based on the guidelines while continuously monitoring patients’ renal function and performing TDM. In addition to the model library developed in this study, Chinese guidelines or limited sampling strategies can be adopted to facilitate precision dosing, ultimately improving both the effectiveness and the safety of PMB therapy (Wang et al., 2020; Liu et al., 2023a). Therefore, conclusions regarding covariate effects and exposure predictions should be interpreted in light of the sample size and clinical representativeness of the source studies. Larger studies may provide more stable and precise estimates of covariate effects, reduce the uncertainty caused by sparse sampling or population heterogeneity, and clarify whether factors such as body weight and renal function should meaningfully influence individualized polymyxin B dosing. In particular, findings from larger prospective cohorts may either strengthen or modify the present conclusions regarding the relationship between covariates and model-predicted exposure.

PMB has traditionally been regarded as less dependent on renal elimination than colistin, and clinical guidelines have not recommended routine dose adjustment based solely on impaired renal function (Tsuji et al., 2019; Zamri et al., 2025). At the same time, several of the included popPK models retained CrCL, blood urea nitrogen, CRRT, or related renal variables as significant covariates (Wang et al., 2021b; Yu et al., 2021; Luo et al., 2022; Wang et al., 2022b; Li et al., 2023). In simulations, models incorporating renal function often predicted higher exposure in patients with reduced renal clearance (Wang et al., 2021b; Yu et al., 2021). These results do not overturn existing guideline recommendations; rather, they indicate that renal function and renal replacement therapy can serve as important markers of altered polymyxin B disposition in certain clinical datasets. Our findings revealed notable differences in drug exposure between patients with normal renal function and those with renal impairment. Furthermore, the model library and accompanying Shiny application enable clinicians and pharmacists to rapidly estimate AUC_ss,24h_ based on patient covariates, thereby facilitating MIPD of polymyxin B in clinical settings (Tam et al., 2020; Liu et al., 2023a).

International consensus guidelines recommend weight-based dosing for polymyxin B (Tsuji et al., 2019). However, among the 22 studies reviewed, only one identified body weight as a significant pharmacokinetic covariate (Wang et al., 2021a). Notably, pharmacokinetic parameters in obese patients did not differ substantially from those in normal-weight patients. In addition, a large international study also failed to retain body weight as a significant covariate (Hanafin et al., 2023). The guideline recommendation for weight-based dosing was derived primarily from a population pharmacokinetic study with a limited sample size, whereas the larger and more recent studies included in this review challenge that recommendation.

The present study developed a parametric popPK model library for PMB to support model informed precision dosing. By integrating published models within a common R framework, the library allows users to simulate different dosing scenarios in clinically relevant patient subgroups and to assess model performance using local data when available. To further facilitate clinical application, we have also built an R Shiny platform based on this model library, which allows clinicians without coding or modeling expertise to conveniently perform simulations and Bayesian estimation for individual exposure prediction. This framework may help researchers compare candidate models, examine factors affecting prediction accuracy, and select an appropriate model for individual exposure prediction. When using the Shiny platform, users are first required to select the most appropriate model based on the actual patient characteristics, then input the corresponding covariates and dosing regimen to perform simulations and predictions. When using the models provided in the framework for simulation and Bayesian estimation, we recommend that users first verify whether the original studies have conducted VPC and shrinkage assessments, as these validations are essential for ensuring the reliability of the outputs. Moreover, model selection and model averaging approaches can also be applied to reduce reliance on a single model and improve the robustness of patient-specific predictions. In addition, the PMB popPK model library can be combined with other R-based pharmacometric tools to support related research, such as clinical trial design optimization using the PopED package (https://andrewover.github.io/PopED/index.html).

Several limitations of this study should be acknowledged. First, given the discrepancies in parameter estimation methods and underlying model assumptions between nonparametric and parametric models, all studies that developed models via nonparametric approaches were excluded during the literature screening process to enhance the comparability of the studies included in this paper. Second, our simulation scenarios did not account for correlations between two or more covariates when multiple covariates were incorporated into a model. Because correlated covariates should be excluded during model development to avoid collinearity, this assumption is not entirely unreasonable. Moreover, this setting ensures the accuracy of pharmacokinetic simulations for a typical subject. Nevertheless, the simulated exposure profiles may not fully reflect the covariate distributions of the study populations in the included studies. Further refinement of the repository with patient level data and newly published reproducible models will help improve its applicability across heterogeneous clinical settings.

## 5 Conclusion

In conclusion, the PMB parametric population pharmacokinetic model library provides a practical framework for model-informed precision dosing and supports systematic comparison of published models across clinically relevant populations. This model library offers a valuable resource to assist clinicians in selecting optimal dosing regimens and conducting effective therapeutic drug monitoring.

## Data Availability

The original contributions presented in the study are included in the article/Supplementary Material, further inquiries can be directed to the corresponding author.
