# Supplementary material for: A population pharmacokinetic model library for polymyxin B: clinical implementation of model-informed precision dosing
Source: Front Pharmacol. 2026 Aug 25;17:1900585. doi: 10.3389/fphar.2026.1900585 (PMC13551480; doi:10.3389/fphar.2026.1900585)
Supplement: Supplementary file 1 [file Supplementaryfile1.docx]

Supplementary Material

# 1 Supplementary Figures and Tables

## 1.1 Supplementary Figures

Figure S1. PRISMA flow diagram of study selection.

The diagram shows the process of literature identification, duplicate removal, title and abstract screening, full-text eligibility assessment, and final inclusion of population pharmacokinetic studies of polymyxin B.

Figure S2. Representative interface of the Shiny-based polymyxin B population pharmacokinetic model library.

The Shiny application integrates the reconstructed polymyxin B population pharmacokinetic models and allows users to select candidate models, input patient-specific covariates, define dosing regimens, and simulate model-predicted exposure to support model-informed precision dosing.

## 1.2 Supplementary Tables

Table S1. Searching strategy.

| PubMed | Search mode | Number |
| --- | --- | --- |
| #1 | ((((Polymyxin B[MeSH Terms]) OR (Polymyxin B)) OR (Polymyxin B Sulfate)) OR (Aerosporin)) | 7764 |
| #2 | (((((((((((((Population Pharmacokinetics[MeSH Terms]) OR (Population Pharmacokinetic)) OR (pharmacokinetic modeling)) OR (nonlinear mixed effect model)) OR (NONMEM)) OR (Pmetrics)) OR (WINNONMIX)) OR (ADAPT)) OR (P-PHARM)) OR (nlmixed)) OR (NLME)) OR (USC*PACK)) OR (MONOLIX)) OR (MRGSOLVE) | 1180030 |
| #3 | #1 AND #2 | 347 |
| Web of Science | Search mode | Number |
| #1 | (((TS=(Polymyxin B)) OR TS=(Polymyxin B Sulfate)) OR TS=(Aerosporin)) | 13320 |
| #2 | ((((((((((((TS=(Population Pharmacokinetics)) OR TS=(Population Pharmacokinetic)) OR TS=(pharmacokinetic modeling)) OR TS=(nonlinear mixed effect model)) OR TS=(NONMEM)) OR TS=(Pmetrics)) OR TS=(WINNONMIX)) OR TS=(ADAPT)) OR TS=(P-PHARM)) OR TS=(nlmixed)) OR TS=(NLME)) OR TS=(USC*PACK)) OR TS=(MONOLIX) OR TS=(MRGSOLVE) | 2520567 |
| #3 | #1 AND #2 | 625 |
| Embase | Search mode | Number |
| #1 | 'Polymyxin B'/exp OR 'Polymyxin B Sulfate' OR 'Aerosporin' | 15368 |
| #2 | 'population pharmacokinetics'/exp OR 'population pharmacokinetic' OR (('population'/exp OR population) AND ('pharmacokinetics'/exp OR pharmacokinetics)) OR (population AND pharmacokinetic) OR (pharmacokinetic AND modeling) OR (nonlinear AND mixed AND efect AND model) OR 'nonmem' OR 'pmetrics' OR 'winnonmix' OR 'adapt' OR 'p pharm' OR 'nlmixed' OR 'nlme' OR 'usc*pack' OR 'monolix' OR 'mrgsolve' | 196233 |
| #3 | #1 AND #2 | 197 |

Table S2. Checklist of information to be included when reporting a clinical pharmacokinetic study.

| **Model ID** | **M1** | **M2** | **M3** | **M4** | **M5** | **M6** | **M7** | **M8** | **M9** | **M10** | **M11** | **M12** | **M13** | **M14** | **M15** | **M16** | **M17** | **M18** | **M19** | **M20** | **M21** | **M22** |
| --- | --- | --- | --- | --- | --- | --- | --- | --- | --- | --- | --- | --- | --- | --- | --- | --- | --- | --- | --- | --- | --- | --- |
| Drug and patient population | 1 | 1 | 1 | 1 | 1 | 1 | 1 | 1 | 1 | 1 | 1 | 1 | 1 | 1 | 1 | 1 | 1 | 1 | 1 | 1 | 1 | 0.5 |
| Name of the drug(s) studied | 1 | 1 | 1 | 1 | 1 | 1 | 1 | 1 | 1 | 1 | 1 | 1 | 1 | 1 | 1 | 1 | 1 | 1 | 1 | 1 | 1 | 1 |
| Population studied | 1 | 1 | 1 | 1 | 1 | 1 | 1 | 1 | 1 | 1 | 1 | 1 | 1 | 1 | 1 | 1 | 1 | 1 | 1 | 1 | 1 | 1 |
| Primary objective | 1 | 1 | 1 | 1 | 1 | 1 | 1 | 1 | 1 | 1 | 1 | 1 | 1 | 1 | 1 | 1 | 1 | 1 | 1 | 1 | 1 | 1 |
| Major findings | 1 | 1 | 1 | 1 | 1 | 1 | 1 | 1 | 1 | 1 | 1 | 1 | 1 | 1 | 1 | 1 | 1 | 1 | 1 | 1 | 1 | 1 |
| Pharmacokinetic data | 1 | 1 | 1 | 1 | 1 | 1 | 1 | 1 | 1 | 1 | 1 | 1 | 1 | 1 | 1 | 1 | 1 | 1 | 1 | 1 | 1 | 1 |
| Study rationale | 1 | 1 | 1 | 1 | 1 | 1 | 1 | 1 | 1 | 1 | 1 | 1 | 1 | 1 | 1 | 1 | 1 | 1 | 1 | 1 | 1 | 1 |
| Specific objectives/hypotheses | 1 | 1 | 1 | 1 | 1 | 1 | 1 | 1 | 1 | 1 | 1 | 1 | 1 | 1 | 1 | 1 | 1 | 1 | 1 | 1 | 1 | 1 |
| Eligibility criteria | 1 | 1 | 1 | 1 | 1 | 1 | 1 | 1 | 1 | 1 | 0 | 1 | 1 | 1 | 1 | 1 | 1 | 1 | 1 | 1 | 1 | 1 |
| Ethic requirement | 1 | 1 | 1 | 1 | 1 | 1 | 1 | 1 | 1 | 1 | 1 | 1 | 1 | 1 | 1 | 1 | 1 | 1 | 1 | 1 | 1 | 1 |
| Administration strategy | 0 | 1 | 0 | 1 | 1 | 0.5 | 1 | 1 | 1 | 0.5 | 1 | 1 | 0.5 | 1 | 0 | 1 | 1 | 1 | 1 | 0.5 | 1 | 1 |
| Covariates | 1 | 1 | 1 | 1 | 1 | 1 | 1 | 1 | 1 | 1 | 1 | 1 | 1 | 1 | 1 | 1 | 1 | 1 | 1 | 1 | 1 | 1 |
| Sampling schedule | 1 | 1 | 1 | 1 | 1 | 1 | 1 | 1 | 1 | 1 | 1 | 1 | 1 | 1 | 1 | 1 | 1 | 1 | 1 | 1 | 1 | 1 |
| Bioanalytical methods | 1 | 1 | 1 | 1 | 1 | 1 | 1 | 1 | 1 | 1 | 1 | 1 | 1 | 1 | 1 | 1 | 1 | 1 | 1 | 1 | 1 | 1 |
| Modeling software | 1 | 1 | 1 | 1 | 1 | 1 | 1 | 1 | 1 | 1 | 1 | 1 | 1 | 1 | 1 | 1 | 1 | 1 | 1 | 1 | 1 | 1 |
| Statistical methods and software | 1 | 1 | 1 | 1 | 1 | 1 | 1 | 1 | 1 | 1 | 1 | 1 | 1 | 1 | 1 | 1 | 1 | 1 | 1 | 1 | 1 | 1 |
| Candidate structural models | 1 | 1 | 1 | 1 | 1 | 1 | 1 | 1 | 1 | 1 | 1 | 1 | 1 | 1 | 1 | 1 | 1 | 1 | 1 | 1 | 1 | 1 |
| Residual error structure | 1 | 1 | 1 | 1 | 1 | 1 | 1 | 1 | 1 | 1 | 1 | 1 | 1 | 1 | 1 | 1 | 1 | 1 | 1 | 1 | 1 | 1 |
| Base model determination and evaluation | 1 | 1 | 1 | 1 | 1 | 1 | 1 | 1 | 1 | 1 | 1 | 1 | 1 | 1 | 1 | 1 | 1 | 1 | 1 | 1 | 1 | 1 |
| Covariates analysis strategy | 1 | 1 | 1 | 1 | 1 | 1 | 1 | 1 | 1 | 1 | 1 | 1 | 1 | 1 | 1 | 1 | 1 | 1 | 1 | 1 | 1 | 0 |
| Final model evaluation | 1 | 1 | 1 | 1 | 1 | 1 | 1 | 1 | 1 | 1 | 0 | 1 | 1 | 0 | 1 | 1 | 1 | 0 | 1 | 1 | 1 | 1 |
| Distribution of individual model parameters | 1 | 1 | 1 | 1 | 1 | 1 | 1 | 1 | 1 | 1 | 1 | 1 | 1 | 1 | 1 | 1 | 1 | 1 | 1 | 1 | 1 | 1 |
| Estimation method(s) used | 1 | 1 | 1 | 1 | 1 | 1 | 1 | 1 | 1 | 1 | 1 | 1 | 1 | 1 | 1 | 1 | 1 | 1 | 1 | 1 | 1 | 1 |
| Population characteristic | 1 | 1 | 1 | 1 | 1 | 1 | 1 | 1 | 1 | 1 | 1 | 1 | 1 | 1 | 1 | 1 | 1 | 1 | 1 | 1 | 1 | 1 |
| Number of subjects and observations | 1 | 1 | 1 | 1 | 1 | 1 | 0.5 | 1 | 1 | 1 | 1 | 1 | 1 | 1 | 1 | 1 | 1 | 1 | 0.5 | 1 | 1 | 1 |
| Schematic of the final model | 0 | 1 | 0 | 0 | 0 | 0 | 1 | 0 | 0 | 0 | 0 | 0 | 0 | 0 | 0 | 0 | 0 | 0 | 1 | 0 | 0 | 0 |
| Table of the final model parameters | 1 | 1 | 1 | 1 | 1 | 1 | 1 | 1 | 1 | 1 | 1 | 1 | 1 | 1 | 1 | 1 | 1 | 1 | 1 | 1 | 1 | 1 |
| Final model evaluation plots | 1 | 1 | 1 | 1 | 1 | 1 | 1 | 1 | 1 | 0 | 1 | 1 | 1 | 1 | 1 | 1 | 1 | 1 | 1 | 1 | 1 | 0 |
| Description of the model-building process | 1 | 1 | 1 | 1 | 1 | 1 | 1 | 1 | 1 | 1 | 1 | 1 | 1 | 1 | 1 | 1 | 1 | 1 | 1 | 1 | 1 | 1 |
| Plot of concentrations vs. time | 0 | 1 | 0 | 0 | 0 | 0 | 1 | 1 | 1 | 0 | 1 | 1 | 0 | 0 | 1 | 1 | 1 | 1 | 1 | 1 | 0 | 1 |
| Study limitations | 1 | 1 | 1 | 1 | 1 | 1 | 1 | 1 | 1 | 1 | 1 | 1 | 1 | 1 | 1 | 1 | 1 | 1 | 0 | 1 | 1 | 1 |
| Study findings | 1 | 1 | 1 | 1 | 1 | 1 | 1 | 1 | 1 | 1 | 1 | 1 | 1 | 1 | 1 | 1 | 1 | 1 | 1 | 1 | 1 | 1 |
| Mean: 93.98 | 90.6 | 100.0 | 90.6 | 93.8 | 93.8 | 92.2 | 98.4 | 96.9 | 96.9 | 89.1 | 90.6 | 96.9 | 92.2 | 90.6 | 93.8 | 96.9 | 96.9 | 93.8 | 95.3 | 95.3 | 93.8 | 89.1 |

Note: M1–M22 correspond to the unified Model IDs (Model 1–Model 22) listed in Tables 1 and 2 of the main manuscript.

Table S3. Study-specific population definitions and eligibility criteria in the included polymyxin B population pharmacokinetic studies.

| **Model ID / Study (year)** | **Population reported in the source study** | **Clinical setting** | **Study-specific eligibility criteria / operational population definition** | **How critical illness was defined** |
| --- | --- | --- | --- | --- |
| Model 1: Yang J. (2025a) | Patients with severe burns (n=53) | Prospective Chinese cohort receiving polymyxin B for infection after severe burn injury. | The source described the population as having severe burns. A numerical total-body-surface-area, burn-depth, or severity-score threshold was not reported in the study description. | Not formally defined using ICU admission or an APACHE II/SOFA threshold. |
| Model 2: Li X.Y. (2025b) | Patients with pneumonia (n=76) | Prospective cohort with pneumonia caused by multidrug-resistant Gram-negative bacteria; nebulized and intravenous polymyxin B administration were evaluated. | Pneumonia and multidrug-resistant Gram-negative infection were the defining clinical features; a common physiologic severity threshold was not specified. | Not labelled or operationally defined as a critically ill cohort. |
| Model 3: Yang J. (2025b) | Critically ill patients with CRO infection (n=95) | Single-centre prospective ICU cohort. Adults (>=18 years) received intravenous polymyxin B for pathogen-confirmed CRO infection and completed at least four consecutive doses, or a loading dose plus at least three consecutive doses. | Excluded missing clinical data, hospitalization <7 days, renal replacement therapy during polymyxin B treatment, and pregnancy. | Operationalized by ICU recruitment and source-study terminology; no independent APACHE II/SOFA eligibility cutoff was reported. |
| Model 4: Wang P. (2024) | Patients with sepsis (n=83) | Prospective Chinese cohort receiving polymyxin B; daily fluid intake was evaluated as a pharmacokinetic covariate. | Sepsis was the source-reported population descriptor. A uniform organ-failure or severity-score eligibility threshold was not reported in the study description. | Not separately defined as critically ill; sepsis status was the defining clinical condition. |
| Model 5: Li X. (2023) | Patients with liver dysfunction (n=138) | Retrospective Chinese cohort receiving polymyxin B; liver dysfunction was evaluated using Child-Pugh classification. | The model included Child-Pugh class and creatinine clearance to characterize hepatic and renal function. The cohort was not selected using a critical-illness score. | Not applicable; the special population was defined by liver dysfunction rather than critical illness. |
| Model 6: Pi M. (2023) | Critically ill patients (n=30) | Prospective cohort recruited through a Department of Critical Care Medicine; 212 polymyxin B samples were included. | The source used the term critically ill and the critical-care setting as the population descriptor; no common physiologic or severity-score threshold was reported as an eligibility definition. | Operationalized by critical-care recruitment; no independent APACHE II/SOFA cutoff reported. |
| Model 7: Yu Z. (2022) | Patients with bloodstream infection (n=9) | Prospective cohort with bloodstream infection caused by carbapenem-resistant Klebsiella pneumoniae. | Microbiologically documented bloodstream infection was the defining feature; no standardized critical-illness criterion was specified. | Not labelled or operationally defined as a critically ill cohort. |
| Model 8: Wang P. (2022a) | Elderly patients (n=23) | Prospective cohort with multidrug-resistant Gram-negative bacterial infection; observed age range was 65-94 years. | Age >=65 years operationalized the elderly population in the analyzed cohort. | Not applicable; age, rather than critical-illness status, defined the population. |
| Model 9: Cai X. (2022) | Lung transplant recipients with pneumonia (n=34) | Prospective adult lung-transplant cohort with confirmed MDR Gram-negative infection. Inclusion required age >=18 years, pneumonia, polymyxin B therapy for >=3 days, and at least one plasma concentration. | Excluded polymyxin B allergy/intolerance and death within 24 hours after starting polymyxin B. | Not applicable; transplant status plus pneumonia defined the population. |
| Model 10: Luo X. (2022) | Critically ill patients with/without CRRT (n=63) | Prospective ICU cohort. Inclusion required age >=18 years, severe infection, confirmed or suspected CRO infection, and intravenous polymyxin B for >=48 hours. | Excluded polymyxin B allergy, unavailable blood samples or death within 48 hours of treatment, and pregnancy. CRRT status defined the comparison groups. | Operationalized by ICU recruitment and severe infection; no APACHE II/SOFA eligibility cutoff was imposed (scores were collected as covariates). |
| Model 11: Wang P. (2022b) | Critically ill patients receiving CVVH (n=53) | Two-hospital observational cohort of adults (>=18 years) receiving intravenous polymyxin B for MDR Gram-negative infection and CRRT/CVVH. | CVVH exposure was directly documented; pharmacokinetic samples were obtained during and, in a subset, after CVVH. | Critical illness was source-reported; CVVH requirement operationalized the special population, but no severity-score cutoff was reported. |
| Model 12: Wang P. (2021a) | Patients with obesity (n=26) | Retrospective cohort with resistant Gram-negative infection receiving polymyxin B for >=3 days. | Obesity was explicitly defined as body mass index >30 kg/m2 (observed range 30.04-40.35 kg/m2). | Not applicable; BMI defined the special population. |
| Model 13: Li Y. (2021) | Renal transplant recipients (n=50) | Prospective renal-transplant cohort receiving polymyxin B; patients covered a broad range of renal function, and some received CRRT during treatment. | Renal transplantation was the defining population characteristic; creatinine clearance and CRRT were recorded to characterize renal function. | Not applicable; transplant status defined the population. |
| Model 14: Wang P. (2021b) | Patients with/without renal insufficiency (n=70) | Retrospective Chinese cohort receiving polymyxin B. | Normal renal function was defined as creatinine clearance >=80 mL/min (n=37); renal insufficiency was defined as creatinine clearance <80 mL/min (n=33). | Not applicable; renal-function category defined the comparison groups. |
| Model 15: Yu X-B. (2021) | Adults with various renal functions (n=32) | Retrospective adult cohort using routine therapeutic-drug-monitoring data during polymyxin B treatment. | Renal function was represented by creatinine clearance and evaluated across clinically relevant ranges; the source did not define the cohort using critical-illness criteria. | Not applicable; renal function was the defining population characteristic. |
| Model 16: Wang P. (2020) | Patients with MDR Gram-negative bacterial infection (n=46) | Prospective Chinese cohort receiving intravenous polymyxin B; rich and sparse samples were used for population PK and limited-sampling analyses. | MDR Gram-negative infection and polymyxin B treatment defined the cohort; no common critical-illness threshold was reported. | Not labelled or operationally defined as a critically ill cohort. |
| Model 17: Xu M. (2024) | Patients receiving ECMO (n=11) | Prospective cohort receiving polymyxin B during extracorporeal membrane oxygenation; different ECMO modes were evaluated. | Receipt and mode of ECMO operationalized the special population. A separate APACHE II/SOFA cutoff was not reported as an eligibility definition. | ECMO use indicates organ-support-level illness, but the source did not provide a uniform severity-score definition of critical illness. |
| Model 18: Ye Q. (2022) | Critically ill patients with/without ECMO (n=44) | Prospective pulmonary and critical-care cohort; eight patients received ECMO and 36 did not. | ECMO support status defined the comparison groups. The source described all patients as critically ill but did not report an independent APACHE II/SOFA threshold for cohort eligibility. | Operationalized by critical-care recruitment; ECMO was an additional organ-support stratifier. |
| Model 19: Liu X. (2021) | Healthy Chinese adults (n=20) | Prospective phase I safety and pharmacokinetic study in healthy adults aged 19-45 years. | Healthy-volunteer screening, rather than a disease or severity definition, determined eligibility. | Not applicable; healthy-volunteer study. |
| Model 20: Hanafin P. (2023) | Hospitalized patients, including RRT (n=142) | Prospective multinational clinical cohort of hospitalized polymyxin B-treated patients from the USA, Singapore, and Brazil; patients receiving renal replacement therapy were included. | Hospitalization and polymyxin B treatment defined the broad cohort. RRT was retained rather than used as an exclusion criterion. | Not restricted to, or uniformly defined as, critically ill patients. |
| Model 21: Tang T. (2023) | Critically ill patients with CRO nosocomial pneumonia (n=105) | Prospective study in two ICUs. Inclusion required age >=18 years; nosocomial pneumonia developing >48 hours after admission; CRO detected in at least two respiratory samples on different days; and intravenous polymyxin B for >3 days. | HAP was defined according to the 2016 IDSA/ATS guideline. Excluded selected pulmonary malignancy/cystic fibrosis, solid-organ or hematopoietic transplantation, hematologic malignancy, and renal replacement therapy. | Operationalized by recruitment from two ICUs; no independent APACHE II/SOFA eligibility cutoff was reported. |
| Model 22: Kubin C. (2018) | Hospitalized adults (n=43) | Retrospective adults (>=18 years) receiving intravenous polymyxin B for suspected or documented Gram-negative infection and undergoing therapeutic drug monitoring. | At least two plasma assays were required. Cystic fibrosis, renal replacement therapy, and other extracorporeal devices at treatment/sampling were excluded; 58% were in an ICU. | Not a uniformly critically ill cohort; ICU residence was present in a subset and was not the defining eligibility criterion. |

Notes: Population terminology follows the source publications. “Not formally defined” indicates that the source used the term critically ill without specifying a common ICU/severity-score eligibility threshold. No retrospective harmonized definition was imposed. NR, not reported; APACHE II, Acute Physiology and Chronic Health Evaluation II; CRO, carbapenem-resistant organism; CRRT, continuous renal replacement therapy; CVVH, continuous venovenous haemofiltration; ECMO, extracorporeal membrane oxygenation; HAP, hospital-acquired pneumonia; ICU, intensive care unit; MDR, multidrug-resistant; RRT, renal replacement therapy; SOFA, Sequential Organ Failure Assessment.

Interpretation: Definitions were not consistent across studies. ICU or critical-care recruitment was commonly used as a pragmatic descriptor, whereas several studies additionally required a specific infection or organ-support modality (CRRT/CVVH or ECMO).

Figure S3. Model-based simulations of AUC_ss,24h_ in patients with varying renal function following a 75 mg intravenous infusion over 1 h.
